# TRPC5 ion channel permeation promotes weight gain in hypercholesterolaemic mice

**DOI:** 10.1038/s41598-018-37299-8

**Published:** 2019-01-28

**Authors:** Baptiste Rode, Nadira Y. Yuldasheva, Paul D. Baxter, Alicia Sedo, Justin F. Ainscough, Michael Shires, Mark T. Kearney, Marc A. Bailey, Stephen B. Wheatcroft, David J. Beech

**Affiliations:** School of Medicine, University of Leeds, Leeds, LS2 9JT UK

## Abstract

Transient Receptor Potential Canonical 5 (TRPC5) is a subunit of a Ca^2+^-permeable non-selective cationic channel which negatively regulates adiponectin but not leptin in mice fed chow diet. Adiponectin is a major anti-inflammatory mediator and so we hypothesized an effect of TRPC5 on the inflammatory condition of atherosclerosis. Atherosclerosis was studied in aorta of *ApoE*^−/−^ mice fed western-style diet. Inhibition of TRPC5 ion permeation was achieved by conditional transgenic expression of a dominant negative ion pore mutant of TRPC5 (DNT5). Gene expression analysis in adipose tissue suggested that DNT5 increases transcript expression for adiponectin while decreasing transcript expression of the inflammatory mediator Tnfα and potentially decreasing Il6, Il1β and Ccl2. Despite these differences there was mild or no reduction in plaque coverage in the aorta. Unexpectedly DNT5 caused highly significant reduction in body weight gain and reduced adipocyte size after 6 and 12 weeks of western-style diet. Steatosis and circulating lipids were unaffected but mild effects on regulators of lipogenesis could not be excluded, as indicated by small reductions in the expression of Srebp1c, Acaca, Scd1. The data suggest that TRPC5 ion channel permeation has little or no effect on atherosclerosis or steatosis but an unexpected major effect on weight gain.

## Introduction

Mammalian Transient Receptor Potential (TRP) proteins are encoded by 28 widely-expressed genes^1^. They cluster together to form tetrameric ion channels with central ion pores which confer membrane permeability to monovalent cations including Na^+^ and usually the divalent cation Ca^2+^ ^2–4^. The tetramers are either homomers or heteromers in which at least one TRP protein is different^2^.

Transient Receptor Potential Canonical 5 (TRPC5) is from the subset of TRP proteins most related to the archetypal *D melanogaster* TRP protein, hence “canonical”^2,5–8^. It was first recognised in 1998 and indicated primarily as a protein of the central nervous system^9^, yet subsequent studies suggested expression and function in a range of tissue and cell types including renal podocytes, vascular smooth muscle cells, endothelial cells and monocytes^10–12^. Its importance in various disease states has been suggested, including depression and kidney disease^13,14^.

In 2012 we reported that TRPC5 is expressed in adipocytes of perivascular fat from patients undergoing coronary artery bypass surgery^15^. We suggested that it generated a constitutively-active channel in heteromers with TRPC1 to permit Ca^2+^ entry into adipocytes, with the downstream consequence of suppressing the generation of adiponectin^15^, a key anti-inflammatory adipokine^16,17^. By mutating an amino acid triplet in TRPC5 which determines ion permeation, we created a dominant negative form of the protein that inhibited Ca^2+^ influx through the channels (DNT5). Conditional expression of DNT5 from a transgene in mice elevated plasma adiponectin, consistent with the idea that the channels suppressed adiponectin. Through an *in vitro* screen of lipids, ω-3 fatty acids were revealed as inhibitors of the channel. When fat was excised from mice expressing DNT5, ω-3 fatty acids had lost their capability to enhance the release of adiponectin, suggesting a mechanism dependent on Ca^2+^ permeation through TRPC5 channels^15^. There were apparently no deleterious effects of expressing DNT5. Overall the data suggested that TRPC5 is part of a Ca^2+^ entry mechanism in adipocytes which is important for the control of the generation or release of adiponectin.

Because adiponectin is a dominant anti-inflammatory mediator, we hypothesized that Ca^2+^ entry through TRPC5 channels might be important in inflammatory diseases such as atherosclerosis. To investigate this hypothesis we transferred DNT5 to a mouse model in which atherosclerosis is accelerated by a combination of *ApoE* gene disruption and western-style diet to elevate plasma cholesterol.

## Results

**Figure 1:**
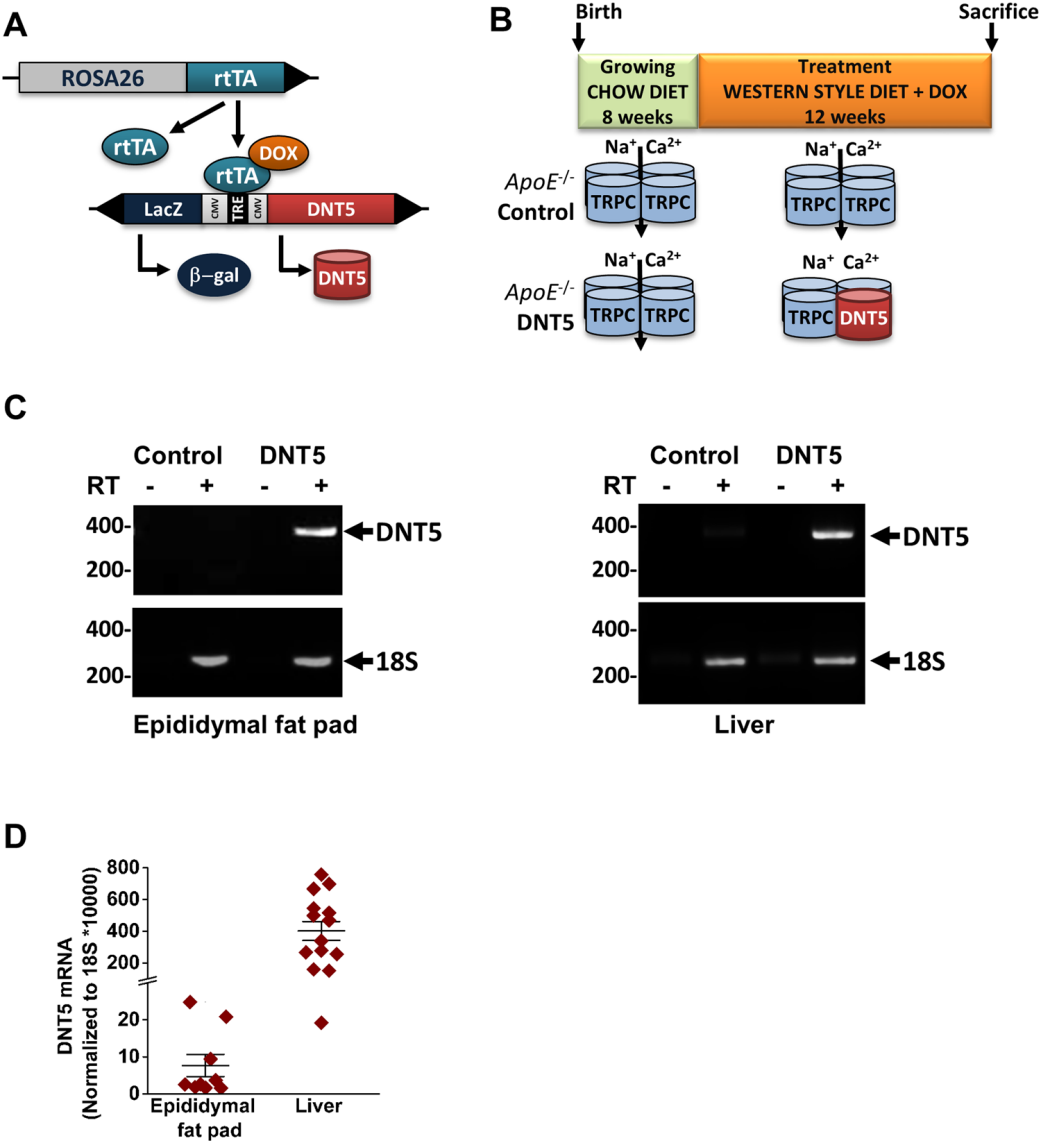
Expression of DNT5 in hypercholesterolaemic mice. (**A**) Schematic of the transgenes regulating the expression of DNT5. DNT5 coding sequence was placed under the control of a Tetracycline Response Element (TRE) that co-regulated the expression of a LacZ reporter. Expression was dependent on the expression of a transactivator (rtTA) from the ROSA26 locus that conferred putatively global expression regulated in a time-controlled manner by addition of doxycycline (DOX) in the drinking water. Only mice with both transgenes expressed DNT5 and were referred as DNT5; non-and single-transgenic mice were the Controls. (**B**) Control and DNT5-expressing mice on *ApoE*^−/−^ background were kept on standard chow diet and did not express DNT5 until 8 weeks of age. During the treatment phase (12 weeks), chow diet was replaced with western-style diet and DOX was given to mice of the two groups, triggering the expression of DNT5 in DNT5 group but not in Controls. All data were from mice which had been provided with western-style diet and DOX for 12 weeks. (**C**) Gel-electrophoresis of end-point RT-PCR performed on epididymial fat pad (left) and liver (right). Results are shown without (−) and with (+) reverse transcription (RT). The sizes of the DNA markers is indicated on the left in base-pairs. The arrows point to the expected PCR product sizes. DNT5 mRNA was detected in tissues from DOX-treated double transgenic mice but not Controls. Gels were cropped for presentation purposes. Full length gels are presented in Supplementary File 1. (**D**) Real-time PCR analysis of the expression of DNT5 mRNA in the epididymal fat pad (N = 9) and liver (N = 14) of DOX-treated double transgenic mice (DNT5 group).

Expression of DNT5 was controlled by the doxycycline (DOX) inducible TET-ON system illustrated in Fig. 1A. All experiments were on *ApoE*^−/−^ male mice on western-style diet from the age of 8 weeks (Fig. 1B). Expression of DNT5 in adipose tissue and liver was detected after induction with DOX (Fig. 1C,D; Supplementary File 1). For every parameter of the present study, observation of values for each genotype did not reveal obvious single-transgene effects (Supplementary File 2). Therefore non-transgenics and single-transgenics were pooled in the Control group as previously^15^. Data were analysed using two different statistical approaches: (1) a Mann-Whitney test comparing the two groups of mouse regardless of the influence of a cage; (2) a linear random effects model considering each cage as a single experiment to enable detection of small effects potentially aliased with cage (see Methods).

### Reduced markers of inflammation in adipose tissue

**Figure 2:**
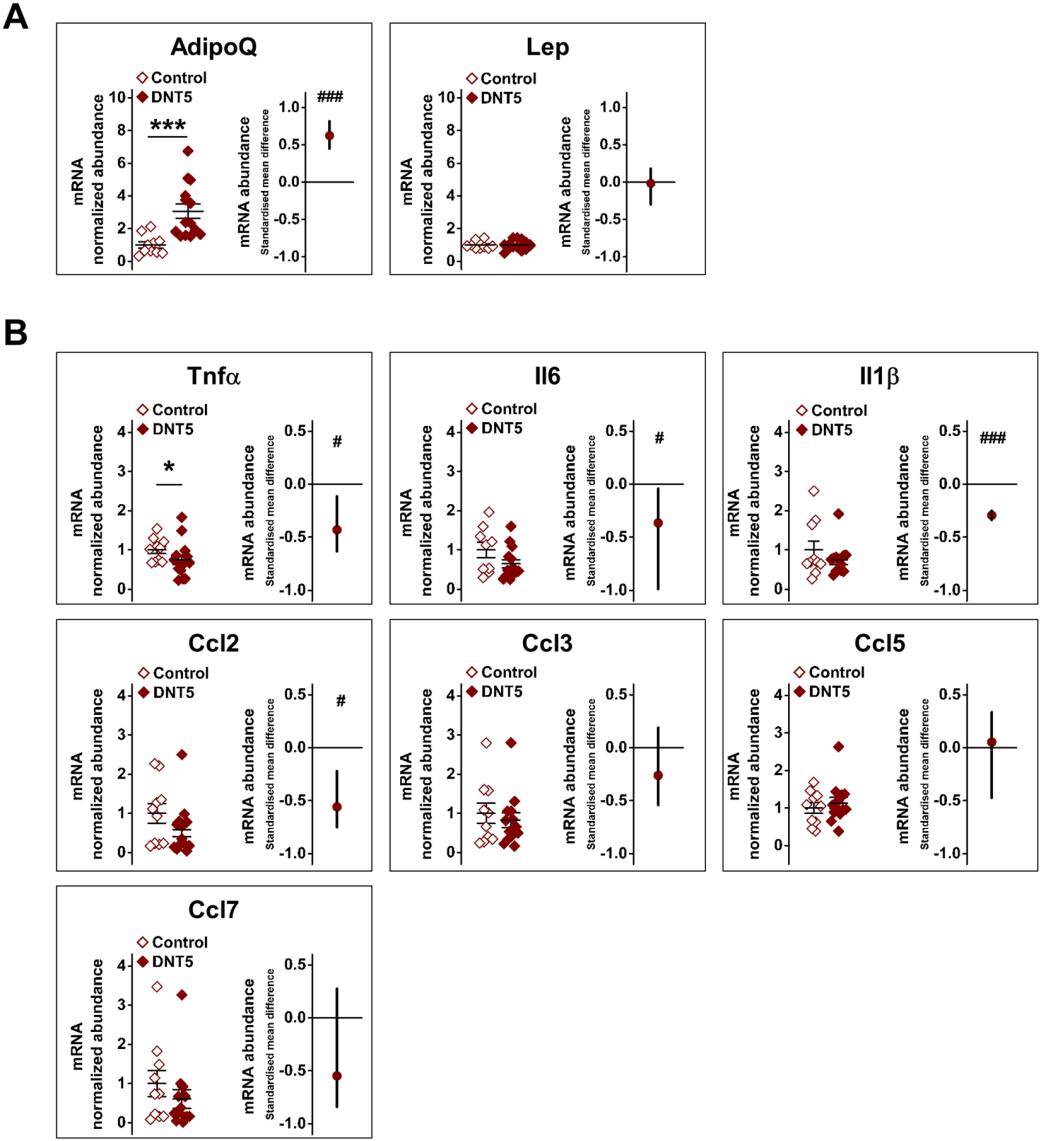
Increased adiponectin and reduced inflammatory gene expression in adipose tissue of DNT5 mice. All data were from quantitative real-time PCR analysis of epididymal fat pad RNA after mice had been provided with western-style diet and DOX for 12 weeks. All mice were *ApoE*^−/−^. (**A**) Comparison of adiponectin (AdipoQ) and leptin (Lep) mRNA abundance between Controls and DNT5. On the left, Mann-Whitney analysis showing mean and s.e.m (N = 10 Control, N = 14 DNT5). On the right, linear random effects model analysis showing the standardised mean difference and confidence intervals (n/N = 8/10 Control, n/N = 8/14 DNT5). Data above the zero line indicate increase in the DNT5 group. (**B**) Comparison of the inflammatory mediators tumour necrosis factor α (Tnfα), interleukin 6 (Il6), interleukin 1β (Il1β) and C-C motif chemokine ligands 2, 3, 5 and 7 (Ccl2, 3, 5 and 7) mRNA abundance between Controls and DNT5. On the left, Mann-Whitney analysis showing mean and s.e.m (N = 10 Control, N = 14 DNT5). On the right, linear random effects model analysis showing the standardised mean difference and confidence intervals. Data below the zero line indicate decrease in the DNT5 group (n/N = 8/10 Control, n/N = 8/14 DNT5).

In line with prior observations on adiponectin and leptin proteins^15^ there was elevated adiponectin transcript in the DNT5 group and no change in leptin (Fig. 2A). Consistent with elevated adiponectin, there was reduced abundance of transcript encoding the key inflammatory mediator including tumour necrosis factor α (Tnfα) and potentially small reductions in interleukin 6 (Il6), interleukin 1β (Il1β) and C-C motif chemokine ligand 2 (Ccl2) (Fig. 2B). The reduction in Il6, Il1β and Ccl2 abundance was statistically significant only when using the linear random effect model, suggesting subtle effect. There was not a general effect on all mediators because expression of C-C motif chemokine ligands 3, 5 and 7 (Ccl3, Ccl5 and Ccl7) was unaffected (Fig. 2B). The data suggested that DNT5 positively impacted adiponectin gene expression and negatively impacted expression of some but not all key pro-inflammatory mediators.

### Mild or no effect on atherosclerosis

**Figure 3:**
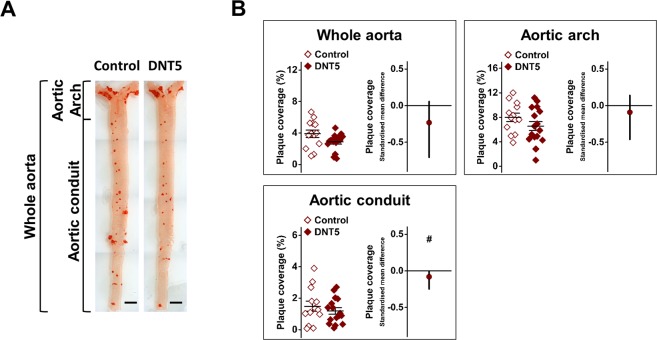
Mild or no effect on atheroma in DNT5 aortic conduit. All data were from mice which had been provided with western-style diet and DOX for 12 weeks. All mice were *ApoE*^−/−^. (**A**) Example aortae from the heart to the iliac bifurcation, opened, and stained with Oil Red O. Scale bar 2 mm. (**B**) Comparison of Oil Red O percentage coverage (Atherosclerosis) between Controls and DNT5 in the whole aorta, aortic arch and aortic conduit as specified in (**A**). On the left, Mann-Whitney analysis showing mean and s.e.m (N = 13 Control, N = 16 DNT5). On the right, linear random effects model analysis showing the standardised mean difference and confidence intervals. Data below the zero line indicate decrease in the DNT5 group (n/N = 10/13 Control, n/N = 10/16 DNT5).

With reciprocal effects of DNT5 on adiponectin and some inflammatory mediators it was anticipated that there may be suppression of atherosclerosis. We quantified plaque coverage in the aorta at the 12-week time point after staining of lipid deposits with Oil Red O (Fig. 3A). Analysis of the whole aorta including the aortic arch did not reveal significant effect of DNT5 but focussed analysis of the aortic conduit suggested that there may have been a small reduction in plaque coverage (Fig. 3A,B). Therefore DNT5 had no major effect on atherosclerosis in the aorta, aside perhaps for a mild region-specific effect.

### Marked reduction in weight gain

**Figure 4:**
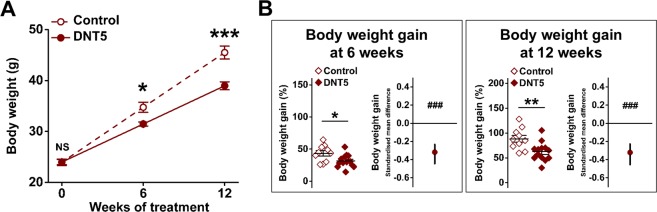
Reduced weight gain in DNT5 mice. All data were from mice which had been provided with western-style diet and DOX for 12 weeks. All mice were *ApoE*^−/−^. (**A**) Body weight of mice from the time of starting western-style diet and DOX treatment at 8 weeks of age Mann-Whitney analysis showing mean and s.e.m (N = 10 Control, N = 13 DNT5). (**B**) Comparison of weight gain between Controls and DNT5. On the left, Mann-Whitney analysis showing mean and s.e.m (N = 10 Control, N = 13 DNT5). On the right, linear random effects model showing the standardised mean difference and confidence intervals. Data below the zero line indicate reduction in the DNT5 group (n/N = 7/10 Control, n/N = 7/13 DNT5).

**Figure 5:**
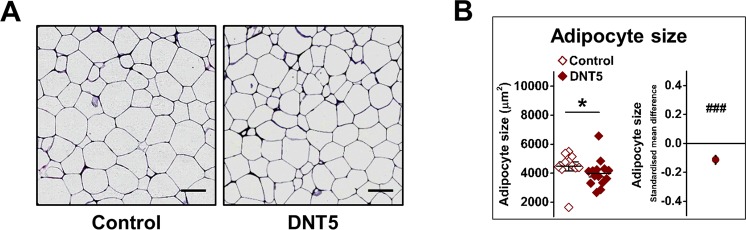
Reduced adipocyte size in DNT5 mice. All data were from mice which had been provided with western-style diet and DOX for 12 weeks. All mice were *ApoE*^−/−^. (**A**) Example of epididymal fat pad sections taken after 12 weeks of treatment and stained with H&E. Scale bar 100 μm. (**B**) Comparison of adipocyte size between Controls and DNT5. On the left, Mann-Whitney analysis showing mean and s.e.m (N = 11 Control, N = 14 DNT5). On the right, linear random effects model showing the standardised mean difference and confidence intervals. Data below the zero line indicate reduction in the DNT5 group (n/N = 8/11 Control, n/N = 8/14 DNT5).

Body weight was measured at 0-, 6- and 12-week time points (Fig. 4A). Mice in both groups appeared in similar health and gained weight. However the DNT5 group unexpectedly displayed lower body weight gain as compared to Controls at both 6- and 12-week time points (Fig. 4A,B). Histological analysis of adipocytes in fat pads revealed that adipocytes were significantly smaller in the DNT5 group (Fig. 5A,B). The data suggested that DNT5 protected against increase in body weight and adipocyte size.

### No effect on steatosis and mild or no effect on lipogenesis regulators

**Figure 6:**
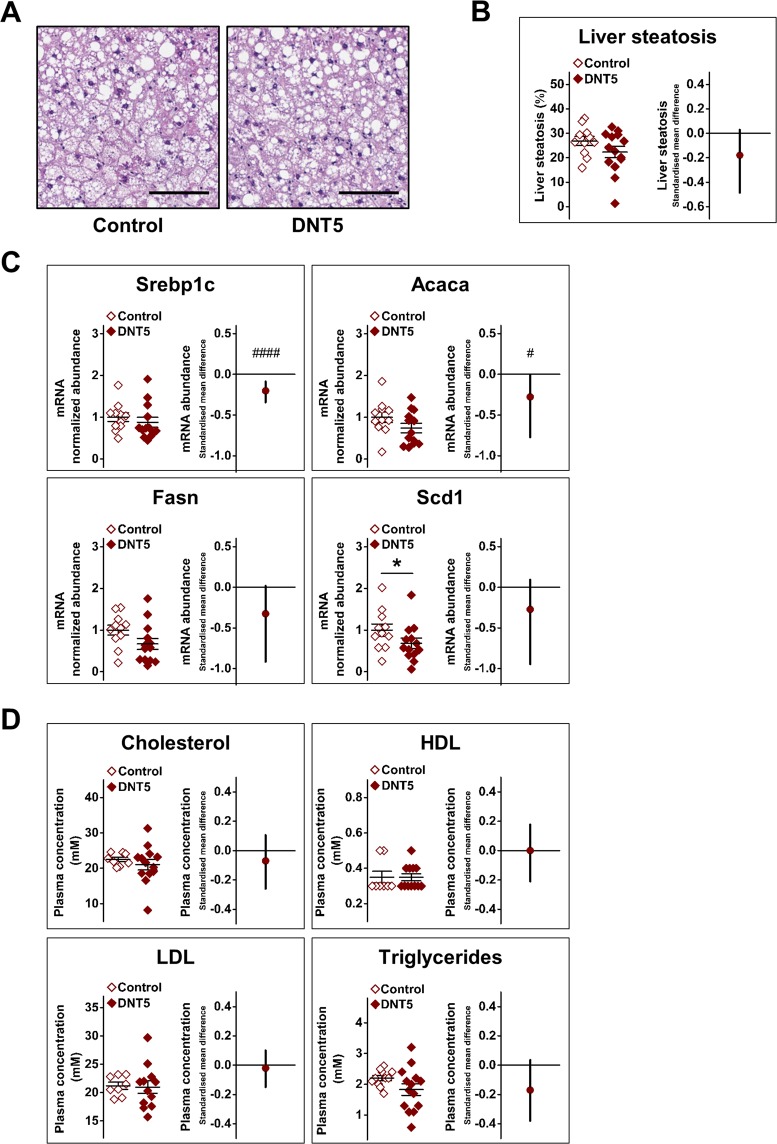
Reduced hepatic gene expression for lipogenesis mediators in DNT5 mice. All data were from mice which had been provided with western-style diet and DOX for 12 weeks. All mice were *ApoE*^−/−^. (**A**) Example liver sections stained with H&E. Scale bar 100 μm. (**B**) Comparison of lipid content (steatosis) between Controls and DNT5. On the left, Mann-Whitney analysis showing mean and s.e.m (N = 11 Control, N = 14 DNT5). On the right, linear random effects model showing the standardised mean difference and confidence intervals. Data below the zero line indicate decrease in the DNT5 group (n/N = 8/11 Control, n/N = 8/13 DNT5). (**C**) All data were from quantitative real-time PCR analysis of liver RNA. Comparison of expression of sterol regulatory element-binding protein 1c gene (Srebp1c), acetyl-CoA carboxylase 1 gene (Acaca), fatty acid synthase gene (Fasn) and stearoyl-CoA desaturase 1 gene (Scd1) mRNA abundance between Controls and DNT5. On the left, Mann-Whitney analysis showing mean and s.e.m (N = 11 Control, N = 14 DNT5). On the right linear random effects model showing the standardised mean difference and confidence intervals. Data below the zero line indicate decrease in the DNT5 group (n/N = 8/11 Control, n/N = 8/13 DNT5). (**D**) Comparison of non-fasting plasma concentrations of cholesterol, high-density lipoprotein (HDL), low-density lipoprotein (LDL) and triglycerides between Controls and DNT5 after 12 weeks of western-style diet and DOX administration. On the left, Mann-Whitney analysis showing mean and s.e.m (N = 11 Control, N = 14 DNT5). On the right, linear random effects model showing the standardised mean difference and confidence intervals (n/N = 8/11 Control, n/N = 8/14 DNT5).

Because of the effect on adiposity we analysed the liver to determine the relevance to steatosis (fatty liver). Histological analysis of the liver suggested no effect of DNT5 on steatosis (Fig. 6A,B). To investigate whether there might be other effects on the liver, we quantified the expression of a key determinant of lipogenesis, sterol regulatory element-binding protein 1c (Srebp1c). The expression of this gene was significantly reduced in the DNT5 group when analysed with the linear random effects model (Fig. 6C). To further explore this effect we investigated the expression of a gene downstream of Srebp1c, the gene encoding acetyl-CoA carboxylase 1 (Acaca) and the fatty acid synthase (Fasn) and the stearoyl-CoA desaturase 1 (Scd1). Expression of Acaca and Scd1 was less in the DNT5 group when using the linear random effects model and the Mann-Whitney test, respectively, consistent with down-regulated Srebp1c gene expression (Fig. 6C). Gene expression of Fasn was not significantly affected (Fig. 6C). Plasma concentrations of cholesterol, high- and low-density lipoproteins (HDL and LDL) and triglycerides were not changed (Fig. 6D). The data suggested that DNT5 had mild effects on hepatic expression of regulators of lipid metabolism but lacked effect on steatosis or plasma lipids.

## Discussion

This study primarily reveals an unexpected positive effect of TRPC5 ion permeation on body weight gain and adipocyte expansion. Consistent with prior work, there was an effect on adiponectin gene expression but changes in the expression of pro-inflammatory mediators were mostly restricted to an effect on Tnfα. Atherosclerosis in the aorta was largely unaffected. A mild effect on hepatic lipogenesis gene expression was apparent but there were no changes in steatosis or plasma lipids.

We analysed our data using two statistical approaches. The Mann-Whitney testing provided global analysis of the two groups of mice and was similar to the approach used in most published studies of this type. This approach allowed us to identify some effects of DNT5 but precluded the observation of effects which were small in size or had high variability. We therefore performed additional analysis by considering each cage of mice as an individual experiment using a linear random effect model. This approach is justified by the fact that behaviour varies from one mouse cage to another, making each cage an individual entity. As expected, differences detected by the Mann-Whitney test (adiponectin and Tnfα gene expression, body weight gain and adipocyte expansion) were also detected by the linear random effect model and are likely to be strongly dependent on TRPC5 channels. Only the hepatic expression of Scd1 was significantly different with the Mann-Whitney test, suggesting this parameter is less dependent on the cage effect. The linear random effect model allowed us to pinpoint other possible effects of DNT5 (adipose tissue expression of Il6, Il1β, Ccl2, atherosclerosis in the artic conduit, hepatic expression of Srebp1c and Acaca). The fact that these parameters show significant difference only with the linear random effect model suggests that they are less dependent on TRPC5 channels or the role of TRPC5 channels might vary from one individual to another, but could contribute to a global beneficial effect of a TRPC5 channel targeting strategy.

DNT5 was conditionally expressed and predicted to achieve effect by incorporating into native channels as they assemble from natively expressed proteins, attenuating the ion permeation in these channels. TRPC5 assembles with TRPC1 and its close relative TRPC4^3,18–20^. Therefore we assume that DNT5 inhibited ion permeation through native channels which involved TRPC1 and TRPC5, the channel suggested to be functionally significant in adipocytes^15^. DNT5 indeed inhibited native TRPC1/TRPC5 heteromers^21^ and to the best of our knowledge there is no evidence for other interactions: DNT5 had no effect on two other TRP channels (TRPM2 and TRPM3) or K_V_ voltage-gated potassium channels which are distantly related to TRPC channels and which assemble similarly as tetramers^15^. Therefore DNT5 has specificity and it is likely that the natural biological rules of these channels ensure specificity.

We previously demonstrated that TRPC5 has a negative effect on adiponectin protein secretion by using DNT5, anti-TRPC5 blocking antibody and linoleic acid (a natural inhibitory of TRPC1/TRPC5 channels), in 3T3-adipocytes and adipose tissue^15^. Here, we provide further information by showing that the effect occurs at the RNA level, suggesting a transcriptional effect or an effect on RNA stability. This effect could be explained by Ca^2+^-dependent regulation of adiponectin gene expression because a Ca^2+^-sensitive transcription factor, namely the nuclear factor of activated T-cells c4 (Nfatc4), was previously shown to be a negative regulator of adiponectin gene expression^22^. TRPC proteins form non-selective cationic channels that also allow Na^+^ and K^+^ flux with possible impact on the membrane potential^2^. It is therefore likely that inhibition of TRPC5 channel permeation has complex consequences due to effects on surrounding voltage-dependent channels in adipocytes but also throughout the body, including the vasculature and the central nervous system.

There are limitations of *ApoE*^−/−^ mice as a model of patients with coronary artery disease: apart from the haemodynamic differences between human and mouse, the hypercholesterolemia is severe and the time for plaque development is short. These mice are however extensively used as a first step to determining relevance in human. In the case of our study, the mice did not develop strongly abundant atherosclerosis as is normally seen because the mice needed to be continuously exposed to DOX, which has anti-atherosclerotic effect^23,24^. We could therefore test for an effect of DNT5, but against a smaller atherosclerotic signal than we ordinarily observe in *ApoE*^−/−^ mice. Nevertheless, atherosclerosis was observed and Control and DNT5 mice received the same DOX, so they both had this background of partial protection against atherosclerosis. This effect is unlikely to be dependent on cholesterol handling as we did not observe difference in circulating levels of cholesterol between the two groups of mice. However, the reduction in inflammation we observe might be beneficial. Plaque formation is complex and other factors such as shear stress and endothelial dysfunction might also have been affected by TRPC5 channels. If there is motivation to further investigate the relationship of TRPC5 to atherosclerosis, alternative genetic approaches would be needed to circumvent the DOX limitation. Alternative models of atherosclerosis could also be explored along with aortic sinus inspection and detailed characterisation of plaque structure.

We considered whether elevated adiponectin expression could explain all or most of the phenotypic changes in the DNT5 group. Reduced adipose tissue inflammation, hepatic Srebp1c expression and atherosclerosis would be consistent with elevated adiponectin^25–27^. However, as we have indicated, some of these effects were mild at best and the effect on inflammation was more specific on Tnfα than might be expected for an adiponectin-mediated phenomenon. Importantly, improvement in glucose homeostasis might arise from inhibition TRPC5 channels as elevation in adiponectin is known to improve insulin sensitivity and glucose tolerance. Continuous administration of sucrose along with DOX in our model might preclude the study of glucose homeostasis in our model but further investigation of the effect of TRPC5 channel inhibition in diabetic models may answer these questions.

It is unclear if the reduced weight gain and reduced adipocyte expansion could be explained by elevated adiponectin. Mice over-expressing adiponectin were found to have lower body weight in models of diet-induced obesity^28,29^ but increased expression of adiponectin in *ob/ob* mice promoted body weight gain^30^ and adiponectin knockout mice had lower body weight gain when fed with high fat diet^31^. The finding of DNT5’s protective effect against weight gain is also superficially contradictory to the finding that neuronal pro-opiomelanocortin-specific disruption of TRPC5 in mice decreased energy expenditure and increased food intake, resulting in elevated body weight^32^. Important differences between the studies could explain the different outcomes, including the genetic background of our mice, the diet and the different technical strategies for interfering with the TRPC5 channels. *Trpc5* knockout mice were found to be protected against hepatomegaly and liver dysfunction in a model of diet-induced cholestasis^33^ and *Trpc1* knockout mice were protected against high fat diet-induced body weight gain^34^. These findings support the idea that the TRPC5/TRPC1 type of ion channel may exacerbate adverse effects in metabolic disorders. We observed an effect on hepatic lipogenesis genes but it was mild, with only a small reduction in expression and not all the genes being affected. This effect could potentially contribute to reduced body weight gain and adipocyte size but is unlikely to be a major contributor of the striking effect on body weight. It is important to note that we used a conditional expression of dominant negative mutant to inhibit the channels which is probably less efficient in inhibiting the channel *in vivo* than a gene knockout but perhaps more likely to be closer to a drug-based strategy. Lipid metabolism might be affected by TRPC5 channels in adipocytes and this might vary from one fad pad to another. Detailed study of lipid handling in abdominal, subcutaneous and perivascular fat, along with brown adipose tissue might help to understand the role of TRPC5 channels in fat accumulation and body weight regulation. It will be important to investigate the mechanisms of the effects on body weight and adipocyte size, including whether global TRPC5 disruption affects food intake, excretion or metabolic rate and whether the effect is mediated peripherally or via the central nervous system. Krout and al have suggested that *Trpc1* knockout mice have reduced food intake^34^ and we have shown that TRPC1/5 channels have physiological function in adipocytes^15^, suggesting multiple roles of TRPC channels in body weight regulation. Despite the mechanism being unknown, our observation of significant reduction in body weight without obvious adverse effect indicates that targeting TRPC5 channels is a potential way to reduce obesity in the context of hypercholesterolaemia.

From a therapeutic perspective the data suggest that inhibitors of TRPC5 channels might beneficially protect against weight gain and potentially have other helpful effects which include reduced adipose tissue inflammation. The study encourages further investigation of the role of these ion channels in weight gain and inflammation and supports the development of new pharmacology for these channels. An important next step forward is the identification of a specific small-molecule inhibitor of the channels which could be administered effectively *in vivo* in mice and other species. After initial limited progress towards such inhibitors^35^, highly potent and specific agents are emerging^13,14,36^. It will be interesting to investigate if inhibitors of this type are protective in cardiovascular and metabolic disease models and if they lack significant unwanted effects on other parameters, which *Trpc5* knockout mouse studies have suggested might occur on joint inflammation^37^ and possibly on blood pressure^38,39^.

## Methods

### Ethical approval

All procedures were approved by the University of Leeds Ethical Review Committee and conducted under UK Home Office licence in accordance with the requirements of the Animals (Scientific Procedures) Act 1986 (UK).

### Transgenic mice

Conditional expression of dominant negative ion pore mutant of TRPC5 (DNT5) in mice has been described previously^15^. Briefly, DNT5 cDNA cloned into the pTRE vector from Clontech was expressed dependent on doxycycline (DOX) regulation of an additional co-expressed transgene encoding reverse tetracycline DOX-regulated transactivator (rtTA) from the ROSA26 locus, designed to confer broad expression across multiple cell types. These mice were crossed with mice homozygous for disruption of *ApoE* (Charles River, Belgium) so that all mice were *ApoE*^−/−^. Primers used for genotyping primer are specified in Table 1. Only male mice were used for experiments. From 8-weeks of age the food for all mice was changed from chow to western-style diet for the next 12 weeks. The diet contained 21% fat from lard supplemented with 0.15% wt/wt cholesterol (#829100, SDS, Witham, Essex, UK). At the same time, all mice received 0.5 mg/mL DOX in the drinking water, sweetened with 2% sucrose. Single transgenics and non-transgenic litter-mates used as Controls also received DOX. Detailed results for each genotype are given in Supplementary File 2. As expected, DOX induced DNT5 expression only in double transgenics.

**Table 1:** Sequence of primers used for genotyping and real-time PCR.

Gene	Application	Forward primer sequence (5′ to 3′)	Reverse primer sequence (5′ to 3′)	Product size (base pairs)
LacZ	Genotyping	AATGGTCTGCTGCTGCTGAAC	GGCTTCATCCACCACATACAG	225
rtTA	Genotyping	TTTCGATCTGGACATGTTGGG	GGCATCGGTAAACATCTGCTC	184
ApoE-WT	Genotyping	GCCTAGCCGAGGGAGAGCCG	TGTGACTTGGGAGCTCTGCAGC	155
ApoE-KO	Genotyping	GCCTAGCCGAGGGAGAGCCG	GCCGCCCCGACTGCATCT	245
18 S	Real-time PCR	GATGCTCTTAGCTGAGTGT	GCTCTGGTCCGTCTTG	233
DNT5	Real-time PCR	CTCTTCAGTCAGCTGCAGCG	TTCTCCGTCTACCGTCAGGG	360
AdipoQ	Real-time PCR	GTATCGCTCAGCGTTC	CGTTGACGTTATCTGCAT	362
Lep	Real-time PCR	CATTTCACACACGCAGT	GGAGGTCTCGGAGATTC	191
Tnfa	Real-time PCR	CCACCACGCTCTTCTGTCTAC	AGGGTCTGGGCCATAGAACT	103
Il6	Real-time PCR	TGATGCACTTGCAGAAAACA	ACCAGAGGAAATTTTCAATAGGC	109
Il1b	Real-time PCR	GCAACTGTTCCTGAACTCAACT	ATCTTTTGGGGTCCGTCAACT	89
Ccl2/Mcp1	Real-time PCR	TGAGTAGGCTGGAGAGCTACAAG	TGTATGTCTGGACCCATTCCTTC	126
Ccl3/Mip1a	Real-time PCR	TGAAACCAGCAGCCTTTGCTC	AGGCATTCAGTTCCAGGTCAGTG	125
Ccl5/Rantes	Real-time PCR	CTCACCATATGGCTCGGACA	ACAAACACGACTGCAAGATTGG	126
Ccl7/Mcp3	Real-time PCR	GCTGCTTTCAGCATCCAAGTG	CCAGGGACACCGACTACTG	135
Srebp1c	Real-time PCR	GGAGCCATGGATTGCACATT	CACTGTCTTGGTTGTTGATGAGCTG	58
Acaca	Real-time PCR	GCCTCTTCCTGACAAACGAG	TGACTGCCGAAACATCTCTG	239
Fasn	Real-time PCR	GCTGCGGAAACTTCAGGAAAT	AGAGACGTGTCACTCCTGGACTT	83
Scd1	Real-time PCR	CCCTGCGGATCTTCCTTATC	TGTGTTTCTGAGAACTTGTGGTG	103

### Tissue collection

Tissue was collected under terminal anaesthesia. Epididymal fat pads and liver were snap frozen on liquid nitrogen and stored at −80 °C. Mice were then perfusion-fixed with phosphate-buffered paraformaldehyde (4% wt/vol, pH 7.2). The entire aortic tree was dissected free of fat and other tissue.

### RNA isolation and real-time RT-PCR

Total RNA was isolated using a standard TriReagent protocol and treated with DNAse (TURBO DNA-free, AM1907M, Ambion). An aliquot was used for cDNA synthesis using a High Capacity RNA-to-cDNA kit (Applied Biosystems, UK) containing Oligo-dT and random primers. Real-time PCR was performed using Roche Fast Start SYBR Green I on a Lightcycler2 with Lightcycler 3.5 software or using Roche 480 SYBR Green I on a Lightcycler480II with Lightcycler 1.5.62 software. DNA amplification was for 35 cycles with an initial 10 min at 95 °C followed by 10 s at 95 °C, 6 s at 55 °C, and 14 s at 72 °C. Primers were used at 0.5 μmole/L. Sequences of PCR primer are specified in Table 1. The specificity of PCR was verified by reactions without RT (-RT) and by melt-curve analysis. PCR cycle crossing-points (CP) were determined by fit-points methodology. Relative abundance of target RNA was calculated from (E_18S_^Cp^)/(E_target_^Cp^). PCR products were electrophoresed on 2% agarose gels containing ethidium bromide. All quantitative PCR reactions were performed in duplicate and the data averaged to generate one value per experiment. On Fig. 1D, data are presented as Relative abundance. On Figs 2–6, data are presented after normalization to the Control group mean value.

### H&E staining

Epididymal fat pad and liver tissues were fixed for 48 hr in 4% PFA at 4 °C prior to processing on a Leica ASP 200 and embedding in CellWax (Cellpath) on a Leica EG1150H embedding station. Sections of 4 μm were cut on a Leica RM2235 microtome onto Plus Frost slides (Solmedia) and allowed to dry at 37 °C overnight prior to staining. Slides were de-waxed in xylene and rehydrated in ethanol. H&E was performed by staining in Mayer’s Haematoxylin for 2 min and eosin for 2 min. Slides were imaged on an Aperio® AT2 (Leica Biosystems) high definition digital pathology slide scanner with a maximal magnification of 20x. Tissue processing and imaging were performed at Section of Pathology and Tumour Biology, Leeds Institute of Cancer and Pathology. Images were analysed with ImageJ software. Adipocyte size was quantified as the cross-section surface area; for each mouse, 2 microscopic fields were analysed and 100 adipocytes per field were measured. Liver steatosis was quantified as the percentage of surface area without staining; for each mouse, 4 microscopic fields were analysed.

### En face analysis of aorta

Aortae were opened longitudinally, stained with Oil Red O and mounted on a coverslip before imaging (Olympus digital camera QICAM on an Olympus SZ61 dissection microscope). Lesion area was analysed using Image-Pro Plus 7.0 (Media Cybernetics, Bethesda, MD) in aortic arch (from the heart to the end of arch curvature), thoracic and abdominal aorta (from arch terminus to iliac bifurcation).

### Plasma lipid analysis

Total blood was collected, without prior fasting, on sodium-citrate from inferior vena cava, centrifuged at 13000 g, 4 °C for 10 minutes; plasma was frozen at −80 °C. Analysis was performed by the Pathology RD Laboratory at Leeds Teaching Hospitals NHS Trust with a Siemens Advia 2400 device.

### Data analysis

Raw data for each parameter are presented in Supplementary File 3. All analyses were performed on a blinded basis. Two statistical approaches were use for data analysis. (1) Data were first analysed for Normality and Equality of variances (Origin Pro). As most of the parameters did not satisfy these criteria, data were analysed with a Mann-Whitney test. The minimum level of statistical significance considered in the study was U < 0.05. Significance is indicated on graphs as follows: ^#^U < 0.05, ^##^U < 0.01 and ^###^U < 0.001; U > 0.05 was considered non-significant. Data are presented as the mean ± s.e.m. (2) We fitted linear random effects models using the statistics package R^40^ and the nlme library^41^. Each outcome measure was modelled against the mouse group (Control or DNT5) and summarised by 95% confidence intervals for the standardised mean difference in outcome measure for DNT5 compared to Control mouse groups. Depending on model convergence, model residual diagnostics and model parameter significance, outcome measures were modelled to either include or not include: (1) a natural log transformation of the outcome measure; (2) a normally distributed random intercept for the cage in which the mice were housed; (3) an identity variance-covariance structure in the normally distributed error term that allowed a separate variance inflation factor for each cage in which the mice were housed. Model output and model structure definitions for each outcome measure are included in the Supplementary File 4. Data are presented as standardised mean difference (red dot) with 95% confidence intervals (vertical black bar), otherwise mentioned. The minimum level of statistical significance considered in the study was p < 0.05. Significance is indicated on graphs as follows: *p < 0.05, **p < 0.01 and ***p < 0.001; p > 0.05 was considered non-significant. The number of independent experiments (cages) is indicated by n and the number of mice by N.

## Supplementary information

Supplementary File 1, 2, 3 and 4

## Data Availability

All data generated or analysed during this study are included in this published article (and its Supplementary Information files). Materials are available on reasonable request.
