# Supplementary material for: TRPC5 ion channel permeation promotes weight gain in hypercholesterolaemic mice
Source: Sci Rep. 2019 Jan 28;9:773. doi: 10.1038/s41598-018-37299-8 (PMC6349875; doi:10.1038/s41598-018-37299-8)
Supplement: Supplementary file 1 — , 2, 3 and 4 [file 41598_2018_37299_MOESM1_ESM.pdf]

SUPPLEMENTARY INFORMATION

ARTICLE TITLE: TRPC5 ion channel permeation promotes weight gain in hypercholesterolaemic mice

AUTHORS: Baptiste Rode PhD, Nadira Y Yuldasheva PhD, Paul D Baxter PhD, Alicia Sedo BSc, Justin F Ainscough PhD, Michael Shires BSc, Mark T Kearney MD, Marc A Bailey MD, Stephen B Wheatcroft MD and David J Beech PhD*

* Corresponding author: Professor David J Beech, Leeds Institute of Cardiovascular and Metabolic Medicine, LIGHT Building, Clarendon Way, School of Medicine, University of Leeds, Leeds LS2 9JT, UK. E-mail d.j.beech@leeds.ac.uk. Telephone +44 (0) 113 3434323.

AUTHOR AFFILIATION: School of Medicine, University of Leeds, Leeds, LS2 9JT, UK

Supplementary File 1. Full-length gels from Figure 1C.

Supplementary File 2. Detailed values for each genotype.

Supplementary File 3. Raw data for each parameter analysed.

Supplementary File 4. Detailed statistical analysis.

SUPPLEMENTARY INFORMATION LEGEND

Supplementary File 1. Full-length gels from Figure 1C. Gel-electrophoresis of end-point RT-PCR performed on epididymal fat pad (left) and liver (right). Results are shown without (-) and with (+) reverse transcription (RT). RT+ reactions were loaded in duplicates for epididymal fat pad. The sizes of the DNA markers (New England Biolabs 100bp ladder) are indicated on the left in base-pairs. The arrows point to the expected PCR product sizes. DNT5 mRNA was detected in tissues from DOX-treated double transgenic mice but not Controls.

Supplementary File 2. Detailed values for each genotype. Individual values for each genotype is shown for each parameter of the present study. Mean value is shown with a horizontal bar. The genotype of each group is indicated in italic on the bottom of each scatter plot: "*DNT5*" refers to the transgene carrying the DNT5 coding sequence under the control of the Tetracycline Response Element, "*rtTA*" refers to the transgene allowing the expression of the rtTA transactivator from the ROSA26 locus. Absence and presence of each transgene are denoted by "-" and "+", respectively. The corresponding groups are indicated underneath.

Supplementary File 1

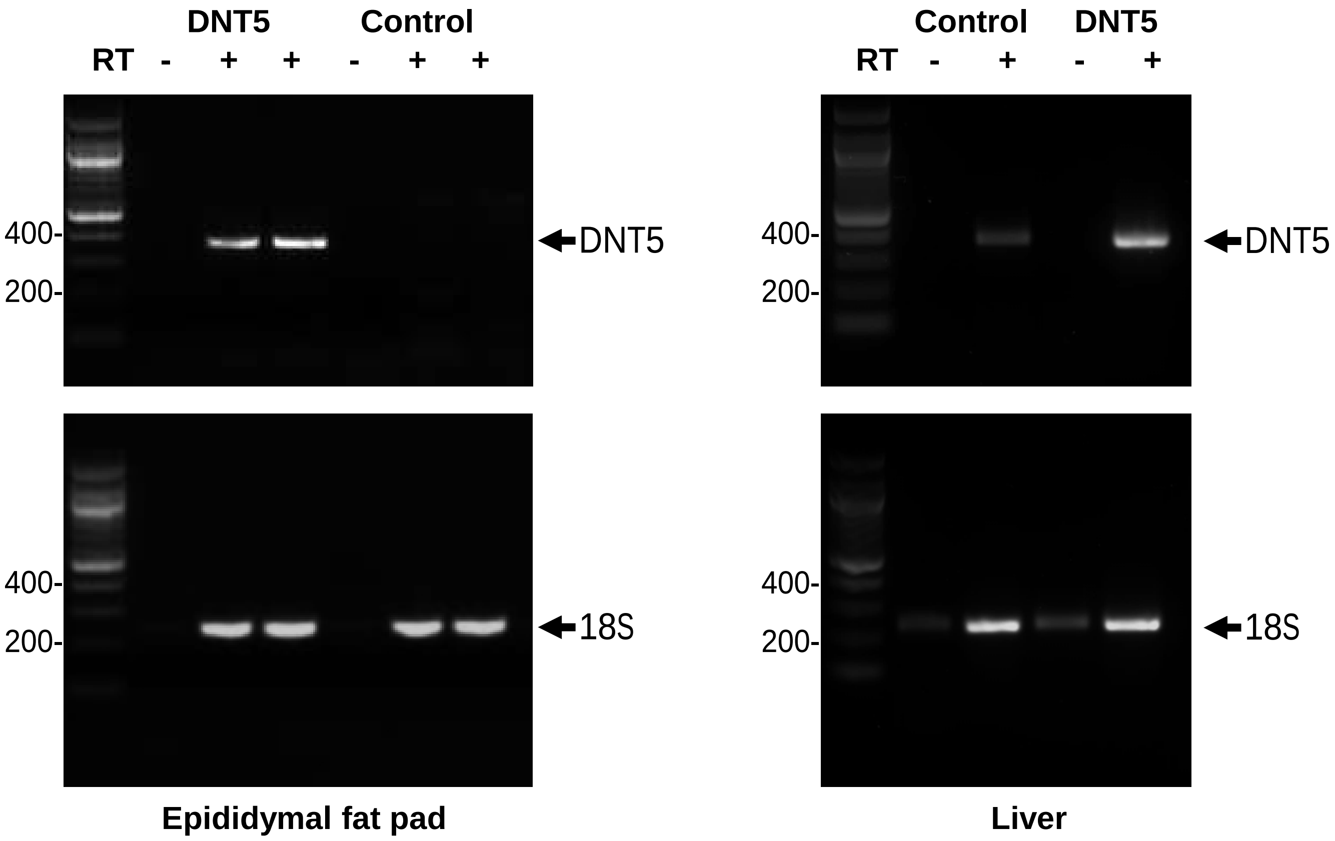

Supplementary File 2 (Part1/2)

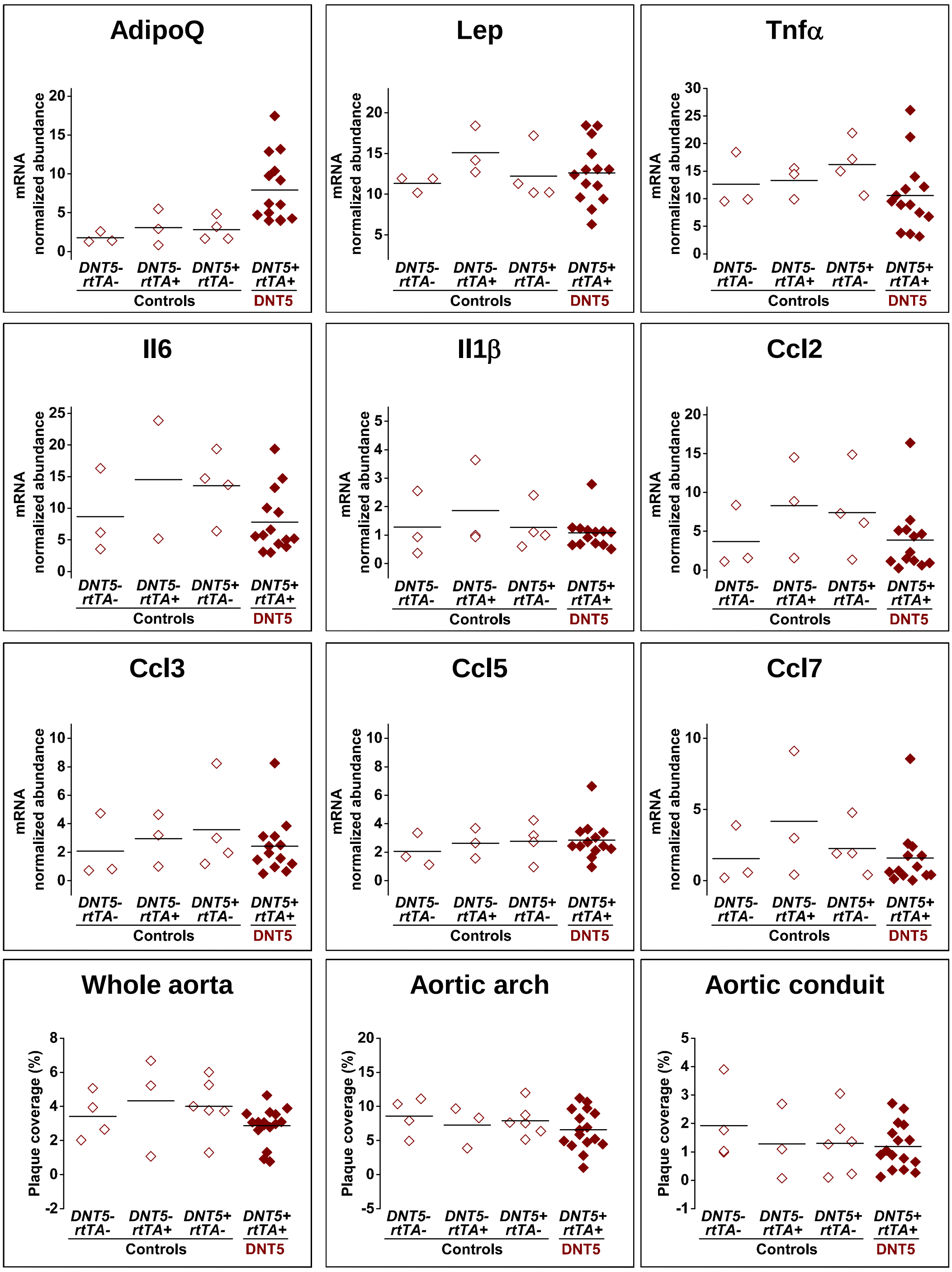

Supplementary File 2 (Part2/2)

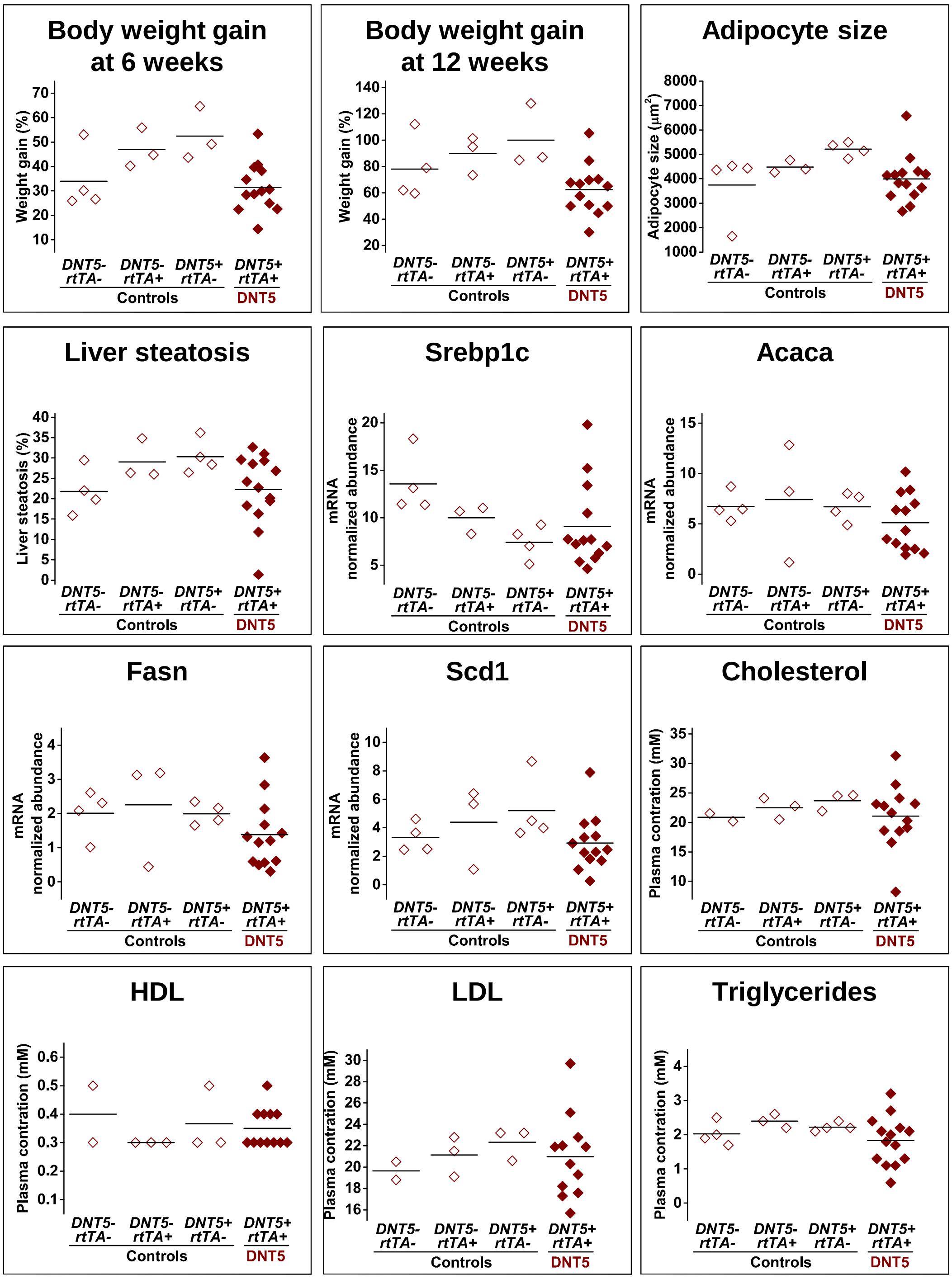

Supplementary File 3

ID	GENOTYPE	GROUP	CAGE	Adipose tissue Adiponectin mRNA relative abundance
49	DNT5+/rtTA-	CT	1	
50	DNT5+/rtTA+	DN	1	
51	DNT5+/rtTA-	CT	2	
52	DNT5+/rtTA+	DN	2	
112	DNT5+/rtTA+	DN	4	4.0
113	DNT5+/rtTA-	CT	4	1.7
118	DNT5+/rtTA+	DN	5	4.1
119	DNT5-/rtTA+	CT	5	2.9
120	DNT5+/rtTA+	DN	5	5.0
121	DNT5+/rtTA+	DN	6	4.0
122	DNT5-/rtTA-	CT	6	2.6
123	DNT5+/rtTA+	DN	6	4.3
124	DNT5+/rtTA+	DN	7	6.0
125	DNT5+/rtTA-	CT	7	3.2
126	DNT5+/rtTA-	CT	7	4.8
127	DNT5+/rtTA+	DN	7	13.2
128	DNT5+/rtTA+	DN	8	4.7
129	DNT5+/rtTA+	DN	8	12.9
130	DNT5-/rtTA+	CT	8	5.5
134	DNT5+/rtTA-	CT	10	1.7
135	DNT5+/rtTA+	DN	10	10.4
136	DNT5+/rtTA+	DN	10	17.5
137	DNT5+/rtTA+	DN	11	9.2
138	DNT5-/rtTA-	CT	11	
139	DNT5-/rtTA-	CT	11	1.4
140	DNT5+/rtTA+	DN	12	6.2
141	DNT5-/rtTA+	CT	12	0.8
142	DNT5-/rtTA-	CT	12	1.3
143	DNT5+/rtTA+	DN	12	9.7

ID	GENOTYPE	GROUP	CAGE	Adipose tissue Leptin mRNA relative abundance
49	DNT5+/rtTA-	CT	1	
50	DNT5+/rtTA+	DN	1	
51	DNT5+/rtTA-	CT	2	
52	DNT5+/rtTA+	DN	2	
112	DNT5+/rtTA+	DN	4	11.0
113	DNT5+/rtTA-	CT	4	10.2
118	DNT5+/rtTA+	DN	5	11.3
119	DNT5-/rtTA+	CT	5	14.2
120	DNT5+/rtTA+	DN	5	18.4
121	DNT5+/rtTA+	DN	6	18.4
122	DNT5-/rtTA-	CT	6	10.2
123	DNT5+/rtTA+	DN	6	8.1
124	DNT5+/rtTA+	DN	7	9.6
125	DNT5+/rtTA-	CT	7	17.2
126	DNT5+/rtTA-	CT	7	11.3
127	DNT5+/rtTA+	DN	7	17.4
128	DNT5+/rtTA+	DN	8	13.1
129	DNT5+/rtTA+	DN	8	15.0
130	DNT5-/rtTA+	CT	8	12.7
134	DNT5+/rtTA-	CT	10	10.2
135	DNT5+/rtTA+	DN	10	9.4
136	DNT5+/rtTA+	DN	10	6.3
137	DNT5+/rtTA+	DN	11	13.0
138	DNT5-/rtTA-	CT	11	
139	DNT5-/rtTA-	CT	11	11.9
140	DNT5+/rtTA+	DN	12	13.0
141	DNT5-/rtTA+	CT	12	18.4
142	DNT5-/rtTA-	CT	12	11.9
143	DNT5+/rtTA+	DN	12	12.4

ID	GENOTYPE	GROUP	CAGE	Adipose tissue TNFα mRNA relative abundance
49	DNT5+/rtTA-	CT	1	
50	DNT5+/rtTA+	DN	1	
51	DNT5+/rtTA-	CT	2	
52	DNT5+/rtTA+	DN	2	
112	DNT5+/rtTA+	DN	4	8.9
113	DNT5+/rtTA-	CT	4	10.6
118	DNT5+/rtTA+	DN	5	8.9
119	DNT5-/rtTA+	CT	5	15.5
120	DNT5+/rtTA+	DN	5	21.2
121	DNT5+/rtTA+	DN	6	14.0
122	DNT5-/rtTA-	CT	6	18.4
123	DNT5+/rtTA+	DN	6	7.5
124	DNT5+/rtTA+	DN	7	3.6
125	DNT5+/rtTA-	CT	7	21.9
126	DNT5+/rtTA-	CT	7	15.0
127	DNT5+/rtTA+	DN	7	11.7
128	DNT5+/rtTA+	DN	8	26.1
129	DNT5+/rtTA+	DN	8	12.2
130	DNT5-/rtTA+	CT	8	14.5
134	DNT5+/rtTA-	CT	10	17.2
135	DNT5+/rtTA+	DN	10	9.5
136	DNT5+/rtTA+	DN	10	6.7
137	DNT5+/rtTA+	DN	11	3.7
138	DNT5-/rtTA-	CT	11	
139	DNT5-/rtTA-	CT	11	9.9
140	DNT5+/rtTA+	DN	12	10.6
141	DNT5-/rtTA+	CT	12	9.9
142	DNT5-/rtTA-	CT	12	9.5
143	DNT5+/rtTA+	DN	12	3.1

ID	GENOTYPE	GROUP	CAGE	Adipose tissue Il6 mRNA relative abundance
49	DNT5+/rtTA-	CT	1	
50	DNT5+/rtTA+	DN	1	
51	DNT5+/rtTA-	CT	2	
52	DNT5+/rtTA+	DN	2	
112	DNT5+/rtTA+	DN	4	9.4
113	DNT5+/rtTA-	CT	4	13.7
118	DNT5+/rtTA+	DN	5	13.2
119	DNT5-/rtTA+	CT	5	23.8
120	DNT5+/rtTA+	DN	5	19.4
121	DNT5+/rtTA+	DN	6	10.0
122	DNT5-/rtTA-	CT	6	16.3
123	DNT5+/rtTA+	DN	6	4.4
124	DNT5+/rtTA+	DN	7	3.0
125	DNT5+/rtTA-	CT	7	19.4
126	DNT5+/rtTA-	CT	7	14.7
127	DNT5+/rtTA+	DN	7	6.6
128	DNT5+/rtTA+	DN	8	14.7
129	DNT5+/rtTA+	DN	8	5.0
130	DNT5-/rtTA+	CT	8	5.2
134	DNT5+/rtTA-	CT	10	6.4
135	DNT5+/rtTA+	DN	10	5.8
136	DNT5+/rtTA+	DN	10	5.2
137	DNT5+/rtTA+	DN	11	3.9
138	DNT5-/rtTA-	CT	11	
139	DNT5-/rtTA-	CT	11	6.2
140	DNT5+/rtTA+	DN	12	5.6
141	DNT5-/rtTA+	CT	12	
142	DNT5-/rtTA-	CT	12	3.5
143	DNT5+/rtTA+	DN	12	3.1

ID	GENOTYPE	GROUP	CAGE	Adipose tissue Il1b mRNA relative abundance
49	DNT5+/rtTA-	CT	1	
50	DNT5+/rtTA+	DN	1	
51	DNT5+/rtTA-	CT	2	
52	DNT5+/rtTA+	DN	2	
112	DNT5+/rtTA+	DN	4	1.11
113	DNT5+/rtTA-	CT	4	1.11
118	DNT5+/rtTA+	DN	5	0.71
119	DNT5-/rtTA+	CT	5	3.64
120	DNT5+/rtTA+	DN	5	1.17
121	DNT5+/rtTA+	DN	6	0.92
122	DNT5-/rtTA-	CT	6	2.55
123	DNT5+/rtTA+	DN	6	1.15
124	DNT5+/rtTA+	DN	7	0.67
125	DNT5+/rtTA-	CT	7	2.40
126	DNT5+/rtTA-	CT	7	1.00
127	DNT5+/rtTA+	DN	7	1.23
128	DNT5+/rtTA+	DN	8	2.79
129	DNT5+/rtTA+	DN	8	1.10
130	DNT5-/rtTA+	CT	8	1.00
134	DNT5+/rtTA-	CT	10	0.60
135	DNT5+/rtTA+	DN	10	0.68
136	DNT5+/rtTA+	DN	10	1.27
137	DNT5+/rtTA+	DN	11	0.51
138	DNT5-/rtTA-	CT	11	
139	DNT5-/rtTA-	CT	11	0.37
140	DNT5+/rtTA+	DN	12	0.66
141	DNT5-/rtTA+	CT	12	0.94
142	DNT5-/rtTA-	CT	12	0.93
143	DNT5+/rtTA+	DN	12	

ID	GENOTYPE	GROUP	CAGE	Adipose tissue Ccl2 mRNA relative abundance
49	DNT5+/rtTA-	CT	1	
50	DNT5+/rtTA+	DN	1	
51	DNT5+/rtTA-	CT	2	
52	DNT5+/rtTA+	DN	2	
112	DNT5+/rtTA+	DN	4	4.35
113	DNT5+/rtTA-	CT	4	6.10
118	DNT5+/rtTA+	DN	5	5.21
119	DNT5-/rtTA+	CT	5	14.50
120	DNT5+/rtTA+	DN	5	6.43
121	DNT5+/rtTA+	DN	6	4.65
122	DNT5-/rtTA-	CT	6	8.37
123	DNT5+/rtTA+	DN	6	2.29
124	DNT5+/rtTA+	DN	7	1.21
125	DNT5+/rtTA-	CT	7	14.87
126	DNT5+/rtTA-	CT	7	7.29
127	DNT5+/rtTA+	DN	7	5.10
128	DNT5+/rtTA+	DN	8	16.37
129	DNT5+/rtTA+	DN	8	1.46
130	DNT5-/rtTA+	CT	8	8.85
134	DNT5+/rtTA-	CT	10	1.35
135	DNT5+/rtTA+	DN	10	0.61
136	DNT5+/rtTA+	DN	10	0.26
137	DNT5+/rtTA+	DN	11	0.91
138	DNT5-/rtTA-	CT	11	
139	DNT5-/rtTA-	CT	11	1.54
140	DNT5+/rtTA+	DN	12	1.15
141	DNT5-/rtTA+	CT	12	1.54
142	DNT5-/rtTA-	CT	12	1.09
143	DNT5+/rtTA+	DN	12	

ID	GENOTYPE	GROUP	CAGE	Adipose tissue Ccl3 mRNA relative abundance
49	DNT5+/rtTA-	CT	1	
50	DNT5+/rtTA+	DN	1	
51	DNT5+/rtTA-	CT	2	
52	DNT5+/rtTA+	DN	2	
112	DNT5+/rtTA+	DN	4	2.50
113	DNT5+/rtTA-	CT	4	1.95
118	DNT5+/rtTA+	DN	5	1.57
119	DNT5-/rtTA+	CT	5	4.64
120	DNT5+/rtTA+	DN	5	3.11
121	DNT5+/rtTA+	DN	6	3.11
122	DNT5-/rtTA-	CT	6	4.73
123	DNT5+/rtTA+	DN	6	1.93
124	DNT5+/rtTA+	DN	7	1.18
125	DNT5+/rtTA-	CT	7	8.23
126	DNT5+/rtTA-	CT	7	2.98
127	DNT5+/rtTA+	DN	7	3.84
128	DNT5+/rtTA+	DN	8	8.25
129	DNT5+/rtTA+	DN	8	2.42
130	DNT5-/rtTA+	CT	8	3.19
134	DNT5+/rtTA-	CT	10	1.18
135	DNT5+/rtTA+	DN	10	0.96
136	DNT5+/rtTA+	DN	10	0.49
137	DNT5+/rtTA+	DN	11	0.66
138	DNT5-/rtTA-	CT	11	
139	DNT5-/rtTA-	CT	11	0.81
140	DNT5+/rtTA+	DN	12	1.46
141	DNT5-/rtTA+	CT	12	1.00
142	DNT5-/rtTA-	CT	12	0.71
143	DNT5+/rtTA+	DN	12	

ID	GENOTYPE	GROUP	CAGE	Adipose tissue Ccl5 mRNA relative abundance
49	DNT5+/rtTA-	CT	1	
50	DNT5+/rtTA+	DN	1	
51	DNT5+/rtTA-	CT	2	
52	DNT5+/rtTA+	DN	2	
112	DNT5+/rtTA+	DN	4	3.03
113	DNT5+/rtTA-	CT	4	3.18
118	DNT5+/rtTA+	DN	5	2.11
119	DNT5-/rtTA+	CT	5	3.67
120	DNT5+/rtTA+	DN	5	3.62
121	DNT5+/rtTA+	DN	6	3.39
122	DNT5-/rtTA-	CT	6	3.35
123	DNT5+/rtTA+	DN	6	2.71
124	DNT5+/rtTA+	DN	7	2.44
125	DNT5+/rtTA-	CT	7	4.24
126	DNT5+/rtTA-	CT	7	2.73
127	DNT5+/rtTA+	DN	7	2.43
128	DNT5+/rtTA+	DN	8	6.63
129	DNT5+/rtTA+	DN	8	2.24
130	DNT5-/rtTA+	CT	8	2.64
134	DNT5+/rtTA-	CT	10	0.96
135	DNT5+/rtTA+	DN	10	2.43
136	DNT5+/rtTA+	DN	10	0.97
137	DNT5+/rtTA+	DN	11	1.63
138	DNT5-/rtTA-	CT	11	
139	DNT5-/rtTA-	CT	11	1.12
140	DNT5+/rtTA+	DN	12	3.45
141	DNT5-/rtTA+	CT	12	1.57
142	DNT5-/rtTA-	CT	12	1.69
143	DNT5+/rtTA+	DN	12	

ID	GENOTYPE	GROUP	CAGE	Adipose tissue Ccl7 mRNA relative abundance
49	DNT5+/rtTA-	CT	1	
50	DNT5+/rtTA+	DN	1	
51	DNT5+/rtTA-	CT	2	
52	DNT5+/rtTA+	DN	2	
112	DNT5+/rtTA+	DN	4	2.41
113	DNT5+/rtTA-	CT	4	1.94
118	DNT5+/rtTA+	DN	5	0.04
119	DNT5-/rtTA+	CT	5	9.11
120	DNT5+/rtTA+	DN	5	2.60
121	DNT5+/rtTA+	DN	6	1.76
122	DNT5-/rtTA-	CT	6	3.89
123	DNT5+/rtTA+	DN	6	0.99
124	DNT5+/rtTA+	DN	7	0.38
125	DNT5+/rtTA-	CT	7	4.79
126	DNT5+/rtTA-	CT	7	1.91
127	DNT5+/rtTA+	DN	7	1.76
128	DNT5+/rtTA+	DN	8	8.56
129	DNT5+/rtTA+	DN	8	0.70
130	DNT5-/rtTA+	CT	8	2.99
134	DNT5+/rtTA-	CT	10	0.42
135	DNT5+/rtTA+	DN	10	0.39
136	DNT5+/rtTA+	DN	10	0.12
137	DNT5+/rtTA+	DN	11	0.41
138	DNT5-/rtTA-	CT	11	
139	DNT5-/rtTA-	CT	11	0.57
140	DNT5+/rtTA+	DN	12	0.61
141	DNT5-/rtTA+	CT	12	0.42
142	DNT5-/rtTA-	CT	12	0.21
143	DNT5+/rtTA+	DN	12	

ID	GENOTYPE	GROUP	CAGE	Whole aorta atherosclerosis area coverage (%)
49	DNT5+/rtTA-	CT	1	1.30
50	DNT5+/rtTA+	DN	1	1.31
51	DNT5+/rtTA-	CT	2	6.01
52	DNT5+/rtTA+	DN	2	0.92
112	DNT5+/rtTA+	DN	4	2.79
113	DNT5+/rtTA-	CT	4	3.76
118	DNT5+/rtTA+	DN	5	3.65
119	DNT5-/rtTA+	CT	5	5.22
120	DNT5+/rtTA+	DN	5	3.06
121	DNT5+/rtTA+	DN	6	2.91
122	DNT5-/rtTA-	CT	6	3.93
123	DNT5+/rtTA+	DN	6	3.54
124	DNT5+/rtTA+	DN	7	3.07
125	DNT5+/rtTA-	CT	7	4.00
126	DNT5+/rtTA-	CT	7	3.74
127	DNT5+/rtTA+	DN	7	3.10
128	DNT5+/rtTA+	DN	8	3.06
129	DNT5+/rtTA+	DN	8	3.89
130	DNT5-/rtTA+	CT	8	1.07
134	DNT5+/rtTA-	CT	10	5.26
135	DNT5+/rtTA+	DN	10	4.64
136	DNT5+/rtTA+	DN	10	0.76
137	DNT5+/rtTA+	DN	11	2.97
138	DNT5-/rtTA-	CT	11	2.65
139	DNT5-/rtTA-	CT	11	2.02
140	DNT5+/rtTA+	DN	12	3.57
141	DNT5-/rtTA+	CT	12	6.68
142	DNT5-/rtTA-	CT	12	5.07
143	DNT5+/rtTA+	DN	12	2.62

ID	GENOTYPE	GROUP	CAGE	Aortic arch atherosclerosis area coverage (%)
49	DNT5+/rtTA-	CT	1	5.14
50	DNT5+/rtTA+	DN	1	4.91
51	DNT5+/rtTA-	CT	2	11.99
52	DNT5+/rtTA+	DN	2	2.82
112	DNT5+/rtTA+	DN	4	9.72
113	DNT5+/rtTA-	CT	4	7.58
118	DNT5+/rtTA+	DN	5	4.77
119	DNT5-/rtTA+	CT	5	8.30
120	DNT5+/rtTA+	DN	5	5.85
121	DNT5+/rtTA+	DN	6	6.93
122	DNT5-/rtTA-	CT	6	7.92
123	DNT5+/rtTA+	DN	6	8.25
124	DNT5+/rtTA+	DN	7	10.64
125	DNT5+/rtTA-	CT	7	7.63
126	DNT5+/rtTA-	CT	7	8.73
127	DNT5+/rtTA+	DN	7	6.55
128	DNT5+/rtTA+	DN	8	5.22
129	DNT5+/rtTA+	DN	8	4.26
130	DNT5-/rtTA+	CT	8	3.87
134	DNT5+/rtTA-	CT	10	6.38
135	DNT5+/rtTA+	DN	10	4.45
136	DNT5+/rtTA+	DN	10	1.00
137	DNT5+/rtTA+	DN	11	8.95
138	DNT5-/rtTA-	CT	11	11.13
139	DNT5-/rtTA-	CT	11	4.94
140	DNT5+/rtTA+	DN	12	9.65
141	DNT5-/rtTA+	CT	12	9.68
142	DNT5-/rtTA-	CT	12	10.34
143	DNT5+/rtTA+	DN	12	11.19

ID	GENOTYPE	GROUP	CAGE	Aortic conduit atherosclerosis area
49	DNT5+/rtTA-	CT	1	0.10
50	DNT5+/rtTA+	DN	1	0.27
51	DNT5+/rtTA-	CT	2	1.81
52	DNT5+/rtTA+	DN	2	0.12
112	DNT5+/rtTA+	DN	4	0.77
113	DNT5+/rtTA-	CT	4	1.36
118	DNT5+/rtTA+	DN	5	1.40
119	DNT5-/rtTA+	CT	5	1.10
120	DNT5+/rtTA+	DN	5	1.96
121	DNT5+/rtTA+	DN	6	2.02
122	DNT5-/rtTA-	CT	6	1.77
123	DNT5+/rtTA+	DN	6	1.66
124	DNT5+/rtTA+	DN	7	0.89
125	DNT5+/rtTA-	CT	7	0.22
126	DNT5+/rtTA-	CT	7	1.27
127	DNT5+/rtTA+	DN	7	0.65
128	DNT5+/rtTA+	DN	8	2.52
129	DNT5+/rtTA+	DN	8	1.05
130	DNT5-/rtTA+	CT	8	0.07
134	DNT5+/rtTA-	CT	10	3.05
135	DNT5+/rtTA+	DN	10	2.70
136	DNT5+/rtTA+	DN	10	0.37
137	DNT5+/rtTA+	DN	11	0.90
138	DNT5-/rtTA-	CT	11	0.98
139	DNT5-/rtTA-	CT	11	1.03
140	DNT5+/rtTA+	DN	12	1.41
141	DNT5-/rtTA+	CT	12	2.69
142	DNT5-/rtTA-	CT	12	3.90
143	DNT5+/rtTA+	DN	12	0.35

ID	GENOTYPE	GROUP	CAGE	Weight gain 6 weeks (%)
49	DNT5+/rtTA-	CT	1	
50	DNT5+/rtTA+	DN	1	
51	DNT5+/rtTA-	CT	2	
52	DNT5+/rtTA+	DN	2	
112	DNT5+/rtTA+	DN	4	
113	DNT5+/rtTA-	CT	4	
118	DNT5+/rtTA+	DN	5	30.0
119	DNT5-/rtTA+	CT	5	55.9
120	DNT5+/rtTA+	DN	5	38.2
121	DNT5+/rtTA+	DN	6	40.7
122	DNT5-/rtTA-	CT	6	53.0
123	DNT5+/rtTA+	DN	6	39.7
124	DNT5+/rtTA+	DN	7	28.7
125	DNT5+/rtTA-	CT	7	64.7
126	DNT5+/rtTA-	CT	7	49.1
127	DNT5+/rtTA+	DN	7	30.7
128	DNT5+/rtTA+	DN	8	53.4
129	DNT5+/rtTA+	DN	8	34.7
130	DNT5-/rtTA+	CT	8	44.8
134	DNT5+/rtTA-	CT	10	43.7
135	DNT5+/rtTA+	DN	10	25.0
136	DNT5+/rtTA+	DN	10	14.4
137	DNT5+/rtTA+	DN	11	28.4
138	DNT5-/rtTA-	CT	11	26.6
139	DNT5-/rtTA-	CT	11	25.9
140	DNT5+/rtTA+	DN	12	22.6
141	DNT5-/rtTA+	CT	12	40.2
142	DNT5-/rtTA-	CT	12	30.2
143	DNT5+/rtTA+	DN	12	22.4

ID	GENOTYPE	GROUP	CAGE	Weight gain 12 weeks (%)
49	DNT5+/rtTA-	CT	1	
50	DNT5+/rtTA+	DN	1	
51	DNT5+/rtTA-	CT	2	
52	DNT5+/rtTA+	DN	2	
112	DNT5+/rtTA+	DN	4	
113	DNT5+/rtTA-	CT	4	
118	DNT5+/rtTA+	DN	5	69.6
119	DNT5-/rtTA+	CT	5	94.9
120	DNT5+/rtTA+	DN	5	66.9
121	DNT5+/rtTA+	DN	6	84.5
122	DNT5-/rtTA-	CT	6	112.1
123	DNT5+/rtTA+	DN	6	70.3
124	DNT5+/rtTA+	DN	7	50.9
125	DNT5+/rtTA-	CT	7	127.9
126	DNT5+/rtTA-	CT	7	87.2
127	DNT5+/rtTA+	DN	7	67.6
128	DNT5+/rtTA+	DN	8	105.3
129	DNT5+/rtTA+	DN	8	57.5
130	DNT5-/rtTA+	CT	8	101.5
134	DNT5+/rtTA-	CT	10	84.9
135	DNT5+/rtTA+	DN	10	44.7
136	DNT5+/rtTA+	DN	10	30.0
137	DNT5+/rtTA+	DN	11	65.1
138	DNT5-/rtTA-	CT	11	59.6
139	DNT5-/rtTA-	CT	11	78.9
140	DNT5+/rtTA+	DN	12	50.0
141	DNT5-/rtTA+	CT	12	73.4
142	DNT5-/rtTA-	CT	12	62.0
143	DNT5+/rtTA+	DN	12	49.8

ID	GENOTYPE	GROUP	CAGE	Adipocyte Area (μm^2)
49	DNT5+/rtTA-	CT	1	
50	DNT5+/rtTA+	DN	1	
51	DNT5+/rtTA-	CT	2	
52	DNT5+/rtTA+	DN	2	
112	DNT5+/rtTA+	DN	4	4847.8
113	DNT5+/rtTA-	CT	4	4819.1
118	DNT5+/rtTA+	DN	5	4243.9
119	DNT5-/rtTA+	CT	5	4764.1
120	DNT5+/rtTA+	DN	5	4304.2
121	DNT5+/rtTA+	DN	6	4156.1
122	DNT5-/rtTA-	CT	6	4528.6
123	DNT5+/rtTA+	DN	6	3780.8
124	DNT5+/rtTA+	DN	7	2862.6
125	DNT5+/rtTA-	CT	7	5498.6
126	DNT5+/rtTA-	CT	7	5367.7
127	DNT5+/rtTA+	DN	7	3822.6
128	DNT5+/rtTA+	DN	8	6574.7
129	DNT5+/rtTA+	DN	8	3349.3
130	DNT5-/rtTA+	CT	8	4262.4
134	DNT5+/rtTA-	CT	10	5143.1
135	DNT5+/rtTA+	DN	10	3305.9
136	DNT5+/rtTA+	DN	10	2666.2
137	DNT5+/rtTA+	DN	11	3634.2
138	DNT5-/rtTA-	CT	11	4357.5
139	DNT5-/rtTA-	CT	11	4433.5
140	DNT5+/rtTA+	DN	12	4192.4
141	DNT5-/rtTA+	CT	12	4396.4
142	DNT5-/rtTA-	CT	12	1645.3
143	DNT5+/rtTA+	DN	12	4131.8

ID	GENOTYPE	GROUP	CAGE	Liver steatosis area coverage (%)
49	DNT5+/rtTA-	CT	1	
50	DNT5+/rtTA+	DN	1	
51	DNT5+/rtTA-	CT	2	
52	DNT5+/rtTA+	DN	2	
112	DNT5+/rtTA+	DN	4	29.4
113	DNT5+/rtTA-	CT	4	36.2
118	DNT5+/rtTA+	DN	5	28.5
119	DNT5-/rtTA+	CT	5	26.0
120	DNT5+/rtTA+	DN	5	31.0
121	DNT5+/rtTA+	DN	6	22.7
122	DNT5-/rtTA-	CT	6	29.4
123	DNT5+/rtTA+	DN	6	16.3
124	DNT5+/rtTA+	DN	7	11.8
125	DNT5+/rtTA-	CT	7	28.4
126	DNT5+/rtTA-	CT	7	30.2
127	DNT5+/rtTA+	DN	7	26.8
128	DNT5+/rtTA+	DN	8	32.6
129	DNT5+/rtTA+	DN	8	24.2
130	DNT5-/rtTA+	CT	8	34.8
134	DNT5+/rtTA-	CT	10	26.4
135	DNT5+/rtTA+	DN	10	20.2
136	DNT5+/rtTA+	DN	10	1.4
137	DNT5+/rtTA+	DN	11	18.3
138	DNT5-/rtTA-	CT	11	19.8
139	DNT5-/rtTA-	CT	11	22.0
140	DNT5+/rtTA+	DN	12	19.4
141	DNT5-/rtTA+	CT	12	26.3
142	DNT5-/rtTA-	CT	12	15.9
143	DNT5+/rtTA+	DN	12	29.6

ID	GENOTYPE	GROUP	CAGE	Liver Srebp1c mRNA relative abundance
49	DNT5+/rtTA-	CT	1	
50	DNT5+/rtTA+	DN	1	
51	DNT5+/rtTA-	CT	2	
52	DNT5+/rtTA+	DN	2	
112	DNT5+/rtTA+	DN	4	4.6
113	DNT5+/rtTA-	CT	4	5.1
118	DNT5+/rtTA+	DN	5	7.7
119	DNT5-/rtTA+	CT	5	11.0
120	DNT5+/rtTA+	DN	5	7.6
121	DNT5+/rtTA+	DN	6	6.3
122	DNT5-/rtTA-	CT	6	11.4
123	DNT5+/rtTA+	DN	6	5.4
124	DNT5+/rtTA+	DN	7	7.2
125	DNT5+/rtTA-	CT	7	9.3
126	DNT5+/rtTA-	CT	7	8.2
127	DNT5+/rtTA+	DN	7	7.0
128	DNT5+/rtTA+	DN	8	13.4
129	DNT5+/rtTA+	DN	8	10.5
130	DNT5-/rtTA+	CT	8	10.7
134	DNT5+/rtTA-	CT	10	7.0
135	DNT5+/rtTA+	DN	10	7.7
136	DNT5+/rtTA+	DN	10	
137	DNT5+/rtTA+	DN	11	19.8
138	DNT5-/rtTA-	CT	11	18.3
139	DNT5-/rtTA-	CT	11	13.1
140	DNT5+/rtTA+	DN	12	15.2
141	DNT5-/rtTA+	CT	12	8.3
142	DNT5-/rtTA-	CT	12	11.4
143	DNT5+/rtTA+	DN	12	5.7

ID	GENOTYPE	GROUP	CAGE	Liver Acaca mRNA relative abundance
49	DNT5+/rtTA-	CT	1	
50	DNT5+/rtTA+	DN	1	
51	DNT5+/rtTA-	CT	2	
52	DNT5+/rtTA+	DN	2	
112	DNT5+/rtTA+	DN	4	2.58
113	DNT5+/rtTA-	CT	4	4.90
118	DNT5+/rtTA+	DN	5	3.06
119	DNT5-/rtTA+	CT	5	8.22
120	DNT5+/rtTA+	DN	5	6.31
121	DNT5+/rtTA+	DN	6	4.34
122	DNT5-/rtTA-	CT	6	6.48
123	DNT5+/rtTA+	DN	6	1.93
124	DNT5+/rtTA+	DN	7	2.50
125	DNT5+/rtTA-	CT	7	7.67
126	DNT5+/rtTA-	CT	7	8.02
127	DNT5+/rtTA+	DN	7	8.36
128	DNT5+/rtTA+	DN	8	10.18
129	DNT5+/rtTA+	DN	8	6.37
130	DNT5-/rtTA+	CT	8	12.84
134	DNT5+/rtTA-	CT	10	6.24
135	DNT5+/rtTA+	DN	10	8.15
136	DNT5+/rtTA+	DN	10	
137	DNT5+/rtTA+	DN	11	7.02
138	DNT5-/rtTA-	CT	11	8.71
139	DNT5-/rtTA-	CT	11	6.39
140	DNT5+/rtTA+	DN	12	3.49
141	DNT5-/rtTA+	CT	12	1.18
142	DNT5-/rtTA-	CT	12	5.30
143	DNT5+/rtTA+	DN	12	2.07

ID	GENOTYPE	GROUP	CAGE	Liver Fasn mRNA relative abundance
49	DNT5+/rtTA-	CT	1	
50	DNT5+/rtTA+	DN	1	
51	DNT5+/rtTA-	CT	2	
52	DNT5+/rtTA+	DN	2	
112	DNT5+/rtTA+	DN	4	0.56
113	DNT5+/rtTA-	CT	4	2.17
118	DNT5+/rtTA+	DN	5	0.59
119	DNT5-/rtTA+	CT	5	3.19
120	DNT5+/rtTA+	DN	5	1.20
121	DNT5+/rtTA+	DN	6	1.67
122	DNT5-/rtTA-	CT	6	2.31
123	DNT5+/rtTA+	DN	6	0.31
124	DNT5+/rtTA+	DN	7	0.50
125	DNT5+/rtTA-	CT	7	2.35
126	DNT5+/rtTA-	CT	7	1.80
127	DNT5+/rtTA+	DN	7	2.14
128	DNT5+/rtTA+	DN	8	2.84
129	DNT5+/rtTA+	DN	8	1.16
130	DNT5-/rtTA+	CT	8	3.13
134	DNT5+/rtTA-	CT	10	1.66
135	DNT5+/rtTA+	DN	10	1.42
136	DNT5+/rtTA+	DN	10	
137	DNT5+/rtTA+	DN	11	3.63
138	DNT5-/rtTA-	CT	11	2.61
139	DNT5-/rtTA-	CT	11	2.08
140	DNT5+/rtTA+	DN	12	1.32
141	DNT5-/rtTA+	CT	12	0.45
142	DNT5-/rtTA-	CT	12	1.02
143	DNT5+/rtTA+	DN	12	0.61

ID	GENOTYPE	GROUP	CAGE	Liver Scd1 mRNA relative abundance
49	DNT5+/rtTA-	CT	1	
50	DNT5+/rtTA+	DN	1	
51	DNT5+/rtTA-	CT	2	
52	DNT5+/rtTA+	DN	2	
112	DNT5+/rtTA+	DN	4	1.80
113	DNT5+/rtTA-	CT	4	3.98
118	DNT5+/rtTA+	DN	5	2.29
119	DNT5-/rtTA+	CT	5	5.68
120	DNT5+/rtTA+	DN	5	2.26
121	DNT5+/rtTA+	DN	6	3.42
122	DNT5-/rtTA-	CT	6	4.61
123	DNT5+/rtTA+	DN	6	0.27
124	DNT5+/rtTA+	DN	7	1.06
125	DNT5+/rtTA-	CT	7	8.67
126	DNT5+/rtTA-	CT	7	4.50
127	DNT5+/rtTA+	DN	7	7.89
128	DNT5+/rtTA+	DN	8	4.48
129	DNT5+/rtTA+	DN	8	2.46
130	DNT5-/rtTA+	CT	8	6.40
134	DNT5+/rtTA-	CT	10	3.65
135	DNT5+/rtTA+	DN	10	3.33
136	DNT5+/rtTA+	DN	10	
137	DNT5+/rtTA+	DN	11	4.30
138	DNT5-/rtTA-	CT	11	3.65
139	DNT5-/rtTA-	CT	11	2.51
140	DNT5+/rtTA+	DN	12	2.92
141	DNT5-/rtTA+	CT	12	1.08
142	DNT5-/rtTA-	CT	12	2.47
143	DNT5+/rtTA+	DN	12	1.69

ID	GENOTYPE	GROUP	CAGE	Cholesterol plasma concentration (mmol/L)
49	DNT5+/rtTA-	CT	1	
50	DNT5+/rtTA+	DN	1	
51	DNT5+/rtTA-	CT	2	
52	DNT5+/rtTA+	DN	2	
112	DNT5+/rtTA+	DN	4	24.1
113	DNT5+/rtTA-	CT	4	
118	DNT5+/rtTA+	DN	5	26.4
119	DNT5-/rtTA+	CT	5	20.5
120	DNT5+/rtTA+	DN	5	31.3
121	DNT5+/rtTA+	DN	6	
122	DNT5-/rtTA-	CT	6	20.2
123	DNT5+/rtTA+	DN	6	18.5
124	DNT5+/rtTA+	DN	7	21.6
125	DNT5+/rtTA-	CT	7	24.5
126	DNT5+/rtTA-	CT	7	21.9
127	DNT5+/rtTA+	DN	7	20.3
128	DNT5+/rtTA+	DN	8	22.8
129	DNT5+/rtTA+	DN	8	23.2
130	DNT5-/rtTA+	CT	8	24.1
134	DNT5+/rtTA-	CT	10	24.6
135	DNT5+/rtTA+	DN	10	23.1
136	DNT5+/rtTA+	DN	10	8.2
137	DNT5+/rtTA+	DN	11	16.6
138	DNT5-/rtTA-	CT	11	21.5
139	DNT5-/rtTA-	CT	11	
140	DNT5+/rtTA+	DN	12	19.1
141	DNT5-/rtTA+	CT	12	22.8
142	DNT5-/rtTA-	CT	12	
143	DNT5+/rtTA+	DN	12	18.6

ID	GENOTYPE	GROUP	CAGE	HDL plasma concentration (mmol/L)
49	DNT5+/rtTA-	CT	1	
50	DNT5+/rtTA+	DN	1	
51	DNT5+/rtTA-	CT	2	
52	DNT5+/rtTA+	DN	2	
112	DNT5+/rtTA+	DN	4	0.4
113	DNT5+/rtTA-	CT	4	
118	DNT5+/rtTA+	DN	5	0.4
119	DNT5-/rtTA+	CT	5	0.3
120	DNT5+/rtTA+	DN	5	0.4
121	DNT5+/rtTA+	DN	6	
122	DNT5-/rtTA-	CT	6	0.5
123	DNT5+/rtTA+	DN	6	0.4
124	DNT5+/rtTA+	DN	7	0.3
125	DNT5+/rtTA-	CT	7	0.3
126	DNT5+/rtTA-	CT	7	0.3
127	DNT5+/rtTA+	DN	7	0.3
128	DNT5+/rtTA+	DN	8	0.3
129	DNT5+/rtTA+	DN	8	0.3
130	DNT5-/rtTA+	CT	8	0.3
134	DNT5+/rtTA-	CT	10	0.5
135	DNT5+/rtTA+	DN	10	0.3
136	DNT5+/rtTA+	DN	10	
137	DNT5+/rtTA+	DN	11	0.3
138	DNT5-/rtTA-	CT	11	0.3
139	DNT5-/rtTA-	CT	11	
140	DNT5+/rtTA+	DN	12	0.3
141	DNT5-/rtTA+	CT	12	0.3
142	DNT5-/rtTA-	CT	12	
143	DNT5+/rtTA+	DN	12	0.5

ID	GENOTYPE	GROUP	CAGE	LDL plasma concentration (mmol/L)
49	DNT5+/rtTA-	CT	1	
50	DNT5+/rtTA+	DN	1	
51	DNT5+/rtTA-	CT	2	
52	DNT5+/rtTA+	DN	2	
112	DNT5+/rtTA+	DN	4	22.8
113	DNT5+/rtTA-	CT	4	
118	DNT5+/rtTA+	DN	5	25.1
119	DNT5-/rtTA+	CT	5	19.1
120	DNT5+/rtTA+	DN	5	29.7
121	DNT5+/rtTA+	DN	6	
122	DNT5-/rtTA-	CT	6	18.8
123	DNT5+/rtTA+	DN	6	17.6
124	DNT5+/rtTA+	DN	7	20.3
125	DNT5+/rtTA-	CT	7	23.2
126	DNT5+/rtTA-	CT	7	20.6
127	DNT5+/rtTA+	DN	7	19.3
128	DNT5+/rtTA+	DN	8	22
129	DNT5+/rtTA+	DN	8	21.9
130	DNT5-/rtTA+	CT	8	22.8
134	DNT5+/rtTA-	CT	10	23.2
135	DNT5+/rtTA+	DN	10	21.9
136	DNT5+/rtTA+	DN	10	
137	DNT5+/rtTA+	DN	11	15.7
138	DNT5-/rtTA-	CT	11	20.5
139	DNT5-/rtTA-	CT	11	
140	DNT5+/rtTA+	DN	12	18.2
141	DNT5-/rtTA+	CT	12	21.5
142	DNT5-/rtTA-	CT	12	
143	DNT5+/rtTA+	DN	12	17.3

ID	GENOTYPE	GROUP	CAGE	Triglycerides plasma concentration (mmol/L)
49	DNT5+/rtTA-	CT	1	
50	DNT5+/rtTA+	DN	1	
51	DNT5+/rtTA-	CT	2	
52	DNT5+/rtTA+	DN	2	
112	DNT5+/rtTA+	DN	4	2
113	DNT5+/rtTA-	CT	4	2.4
118	DNT5+/rtTA+	DN	5	2.1
119	DNT5-/rtTA+	CT	5	2.6
120	DNT5+/rtTA+	DN	5	2.7
121	DNT5+/rtTA+	DN	6	3.2
122	DNT5-/rtTA-	CT	6	2
123	DNT5+/rtTA+	DN	6	1.1
124	DNT5+/rtTA+	DN	7	2.2
125	DNT5+/rtTA-	CT	7	2.2
126	DNT5+/rtTA-	CT	7	2.2
127	DNT5+/rtTA+	DN	7	1.7
128	DNT5+/rtTA+	DN	8	1.1
129	DNT5+/rtTA+	DN	8	2.4
130	DNT5-/rtTA+	CT	8	2.2
134	DNT5+/rtTA-	CT	10	2.1
135	DNT5+/rtTA+	DN	10	2.1
136	DNT5+/rtTA+	DN	10	0.6
137	DNT5+/rtTA+	DN	11	1.3
138	DNT5-/rtTA-	CT	11	1.7
139	DNT5-/rtTA-	CT	11	2.5
140	DNT5+/rtTA+	DN	12	1.3
141	DNT5-/rtTA+	CT	12	2.4
142	DNT5-/rtTA-	CT	12	1.9
143	DNT5+/rtTA+	DN	12	1.8

Supplementary File 4

Adipose tissue Adiponectin mRNA

- Log transformed outcome
- Separate variance for each cage

```
lme(log(MEASURMENTS)~as.factor(GROUP),na.action=na.exclude,random=~1|CAGE,weights=varIdent(form=~1|CAGE))

Linear mixed-effects model fit by REML

       AIC      BIC    logLik
  54.23618 66.23765 -16.11809

Random effects:
 Formula: ~1 | CAGE
        (Intercept) Residual
StdDev:  0.05721544 0.327978

Variance function:
 Structure: Different standard deviations per stratum
 Formula: ~1 | CAGE
 Parameter estimates:
        4         5         6         7         8        10        11        12
1.0000000 0.3108447 0.1478915 1.9500141 2.2698978 2.9893407 2.1503730 2.5430880
Fixed effects: log(MEASURMENTS) ~ as.factor(GROUP)
                       Value  Std.Error DF   t-value p-value
(Intercept)        0.9914514 0.05880142 15 16.861011       0
as.factor(GROUP)DN 0.4837610 0.05271480 15  9.176948       0
 Correlation:
                   (Intr)
as.factor(GROUP)DN -0.585

Standardized Within-Group Residuals:
       Min         Q1        Med         Q3        Max
-1.4517986 -0.5715181  0.4326466  0.9476340  1.7014354

Number of Observations: 24
Number of Groups: 8
```

Adipose tissue Leptin mRNA

- No random intercept

```
lm(MEASURMENTS~as.factor(GROUP),na.action=na.exclude)


Call:
lm(formula = MEASURMENTS ~ as.factor(GROUP), data = baptiste,
    na.action = na.exclude)

Residuals:
   Min     1Q Median     3Q    Max
-6.300 -2.620 -0.560  1.635  5.800

Coefficients:
                   Estimate Std. Error t value Pr(>|t|)
(Intercept)          12.820      1.081  11.862 4.96e-11 ***
as.factor(GROUP)DN   -0.220      1.415  -0.155    0.878
---
Signif. codes:  0 '***' 0.001 '**' 0.01 '*' 0.05 '.' 0.1 ' ' 1

Residual standard error: 3.418 on 22 degrees of freedom
  (11 observations deleted due to missingness)
Multiple R-squared:  0.001098,  Adjusted R-squared:  -0.04431
F-statistic: 0.02417 on 1 and 22 DF,  p-value: 0.8779
```

Adipose Tissue Tnfα mRNA

- Log transformed outcome

```
lme(log(MEASURMENTS)~as.factor(GROUP),na.action=na.exclude,random=~1|CAGE)
Linear mixed-effects model fit by REML

       AIC      BIC   logLik
  53.68639 58.39861 -22.8432

Random effects:
 Formula: ~1 | CAGE
        (Intercept)  Residual
StdDev:    0.247604 0.5189493

Fixed effects: log(MEASURMENTS) ~ as.factor(GROUP)
                        Value Std.Error DF   t-value p-value
(Intercept)         2.7541954 0.1725478 16 15.961926  0.0000
as.factor(GROUP)DN -0.5628675 0.2085491 16 -2.698968  0.0158
 Correlation:
                   (Intr)
as.factor(GROUP)DN -0.644

Standardized Within-Group Residuals:
       Min         Q1        Med         Q3        Max
-1.6725677 -0.4910287 -0.1186797  0.5136341  2.1121970

Number of Observations: 26
Number of Groups: 9
```

Adipose Tissue Il6 mRNA

```
lme(MEASURMENTS~as.factor(GROUP),na.action=na.exclude,random=~1|CAGE)
Linear mixed-effects model fit by REML

       AIC      BIC    logLik
  153.7599 158.3019 -72.87997

Random effects:
 Formula: ~1 | CAGE
        (Intercept) Residual
StdDev:    3.762222 4.232612

Fixed effects: MEASURMENTS ~ as.factor(GROUP)
                       Value Std.Error DF   t-value p-value
(Intercept)        12.248834  1.806212 15  6.781502  0.0000
as.factor(GROUP)DN -4.481659  1.783115 15 -2.513387  0.0239
 Correlation:
                   (Intr)
as.factor(GROUP)DN -0.539

Standardized Within-Group Residuals:
        Min          Q1         Med          Q3         Max
-1.50567381 -0.49408593 -0.03143692  0.39807985  1.79764299

Number of Observations: 25
Number of Groups: 9
```

Adipose Tissue Il1β mRNA

- Log transformed outcome
- Separate variance for each cage

```
lme(log(MEASURMENTS)~as.factor(GROUP),na.action=na.exclude,random=~1|CAGE,weights=varIdent(for
m=~1|CAGE))

Linear mixed-effects model fit by REML


       AIC      BIC    logLik

  51.50191 65.12784 -13.75095


Random effects:

 Formula: ~1 | CAGE

        (Intercept)  Residual

StdDev:   0.2203154 0.2179007


Variance function:

 Structure: Different standard deviations per stratum

 Formula: ~1 | CAGE

 Parameter estimates:

         4          5          6          7          8          9         10         11
12

1.00000000 2.83543986 1.59626891 1.93602280 3.03493341 1.01670964 2.35663960 4.22466494
0.03467693

Fixed effects: log(MEASURMENTS) ~ as.factor(GROUP)

                        Value  Std.Error DF   t-value p-value

(Intercept)         0.2613991 0.10525641 15   2.48345  0.0253

as.factor(GROUP)DN -0.3480921 0.00924146 15 -37.66634  0.0000

 Correlation:

                   (Intr)

as.factor(GROUP)DN -0.039


Standardized Within-Group Residuals:

        Min          Q1         Med          Q3         Max

-1.33165049 -0.56574422 -0.03472342  0.80643271  1.55250694


Number of Observations: 25

Number of Groups: 9
```

<u>Adipose Tissue Ccl2 mRNA</u>

- Log transformed outcome

```
lme(log(MEASURMENTS)~as.factor(GROUP),na.action=na.exclude,random=~1|CAGE)
Linear mixed-effects model fit by REML

      AIC      BIC    logLik
  71.2721 75.81408 -31.63605

Random effects:
 Formula: ~1 | CAGE
        (Intercept)  Residual
StdDev:    0.865878 0.6270326

Fixed effects: log(MEASURMENTS) ~ as.factor(GROUP)
                        Value Std.Error DF   t-value p-value
(Intercept)         1.5810537 0.3433604 15  4.604647  0.0003
as.factor(GROUP)DN -0.8187237 0.2673886 15 -3.061924  0.0079
 Correlation:
                   (Intr)
as.factor(GROUP)DN -0.392

Standardized Within-Group Residuals:
        Min          Q1         Med          Q3         Max
-1.62607729 -0.35049144  0.02538033  0.35756435  2.22361879

Number of Observations: 25
Number of Groups: 9
```

Adipose tissue Ccl3 mRNA

- Log transformed outcome

```
lme(log(MEASURMENTS)~as.factor(GROUP),na.action=na.exclude,random=~1|CAGE)
Linear mixed-effects model fit by REML

      AIC      BIC    logLik
  61.5593 66.10128 -26.77965

Random effects:
 Formula: ~1 | CAGE
        (Intercept)  Residual
StdDev:   0.6421199 0.5256003

Fixed effects: log(MEASURMENTS) ~ as.factor(GROUP)
                        Value Std.Error DF   t-value p-value
(Intercept)         0.9059330 0.2646800 15  3.422749  0.0038
as.factor(GROUP)DN -0.3028831 0.2234118 15 -1.355716  0.1953
 Correlation:
                   (Intr)
as.factor(GROUP)DN -0.426

Standardized Within-Group Residuals:
         Min           Q1          Med           Q3          Max
-1.522076562 -0.536108186 -0.008631966  0.593118802  1.808921007

Number of Observations: 25
Number of Groups: 9
```

Adipose tissue Ccl5 mRNA

```
lme(MEASURMENTS~as.factor(GROUP),na.action=na.exclude,random=~1|CAGE)
Linear mixed-effects model fit by REML

       AIC      BIC    logLik
  89.37191 93.91388 -40.68595

Random effects:
 Formula: ~1 | CAGE
        (Intercept) Residual
StdDev:   0.4870267 1.192009

Fixed effects: MEASURMENTS ~ as.factor(GROUP)
                       Value Std.Error DF  t-value p-value
(Intercept)        2.7151350 0.3834892 15 7.080082  0.0000
as.factor(GROUP)DN 0.1428942 0.4873623 15 0.293199  0.7734
 Correlation:
                   (Intr)
as.factor(GROUP)DN -0.654

Standardized Within-Group Residuals:
        Min          Q1         Med          Q3         Max
-1.20815645 -0.71751485 -0.06598957  0.43911221  2.87451011

Number of Observations: 25
Number of Groups: 9
```

Adipose tissue Ccl7 mRNA

- Log transformed outcome

```
lme(log(MEASURMENTS)~as.factor(GROUP),na.action=na.exclude,random=~1|CAGE)
Linear mixed-effects model fit by REML

       AIC     BIC    logLik
  89.56963 94.1116 -40.78481

Random effects:
 Formula: ~1 | CAGE
        (Intercept) Residual
StdDev:   0.5189745 1.188207

Fixed effects: log(MEASURMENTS) ~ as.factor(GROUP)
                        Value Std.Error DF   t-value p-value
(Intercept)         0.4811997 0.3874108 15  1.242091  0.2333
as.factor(GROUP)DN -0.7951036 0.4868322 15 -1.633219  0.1232
 Correlation:
                   (Intr)
as.factor(GROUP)DN -0.646

Standardized Within-Group Residuals:
        Min          Q1         Med          Q3         Max
-2.45429421 -0.68462015  0.04534429  0.52957325  1.76155561

Number of Observations: 25
Number of Groups: 9
```

Atherosclerosis – Whole aorta

```
lme(MEASURMENTS~as.factor(GROUP),na.action=na.exclude,random=~1|CAGE)
Linear mixed-effects model fit by REML

       AIC      BIC   logLik
  137.3486 143.3346 -64.6743

Random effects:
 Formula: ~1 | CAGE
        (Intercept) Residual
StdDev:   0.8535712 1.391928

Fixed effects: MEASURMENTS ~ as.factor(GROUP)
                       Value Std.Error DF   t-value p-value
(Intercept)         3.429986 0.4018913 21  8.534611  0.0000
as.factor(GROUP)DN -0.793874 0.4975658 21 -1.595516  0.1255
 Correlation:
                   (Intr)
as.factor(GROUP)DN -0.546

Standardized Within-Group Residuals:
       Min         Q1        Med         Q3        Max
-1.6088751 -0.5122920  0.0443579  0.4559438  1.7203455

Number of Observations: 35
Number of Groups: 13
```

Atherosclerosis – Aortic arch

```
lme(MEASURMENTS~as.factor(GROUP),na.action=na.exclude,random=~1|CAGE)
Linear mixed-effects model fit by REML

       AIC      BIC    logLik
  176.4626 182.4486 -84.23128

Random effects:
 Formula: ~1 | CAGE
        (Intercept) Residual
StdDev:     1.84959 2.407985

Fixed effects: MEASURMENTS ~ as.factor(GROUP)
                       Value Std.Error DF   t-value p-value
(Intercept)         6.882241 0.7625181 21  9.025675  0.0000
as.factor(GROUP)DN -0.626173 0.8723517 21 -0.717799  0.4808
 Correlation:
                   (Intr)
as.factor(GROUP)DN -0.5

Standardized Within-Group Residuals:
        Min          Q1         Med          Q3         Max
-1.72605497 -0.39237228 -0.04790167  0.56550442  1.93328981

Number of Observations: 35
Number of Groups: 13
```

Atherosclerosis – Aortic Conduit

- Separate variance for each cage

```
lme(MEASURMENTS~as.factor(GROUP),na.action=na.exclude,random=~1|CAGE,weights=varIdent(form=~1|CAGE))

Linear mixed-effects model fit by REML

       AIC      BIC    logLik
  103.0166 126.9607 -35.50829

Random effects:
 Formula: ~1 | CAGE
        (Intercept)  Residual
StdDev:   0.6754179 0.1922569

Variance function:
 Structure: Different standard deviations per stratum
 Formula: ~1 | CAGE
 Parameter estimates:
        1         2         4         5         6         7         8         9        10
11        12        13        14
1.0000000 4.8726024 1.7466658 2.4423519 1.0661179 2.2941552 5.9888016 0.7913750 7.2411049
0.1830296 7.7441739 2.3529391 0.8158550

Fixed effects: MEASURMENTS ~ as.factor(GROUP)
                        Value  Std.Error DF   t-value p-value
(Intercept)         1.1660331 0.21175552 21  5.506506  0.0000
as.factor(GROUP)DN -0.0936825 0.04138925 21 -2.263451  0.0343
 Correlation:
                   (Intr)
as.factor(GROUP)DN -0.077

Standardized Within-Group Residuals:
        Min          Q1         Med          Q3         Max
-1.40383817 -0.76777524 -0.06686094  0.79237245  1.54262569

Number of Observations: 35
Number of Groups: 13
```

Weight Gain Week 6

- Separate variance for each cage

```
lme(MEASURMENTS~as.factor(GROUP),na.action=na.exclude,random=~1|CAGE,weights=varIdent(form=~1|
CAGE))

Linear mixed-effects model fit by REML

       AIC      BIC    logLik
  226.9532 243.7991 -100.4766

Random effects:
 Formula: ~1 | CAGE
        (Intercept) Residual
StdDev:    10.42275 6.598615

Variance function:
 Structure: Different standard deviations per stratum
 Formula: ~1 | CAGE
 Parameter estimates:
        5         6         7         8         9        10        11        12        13
14
1.0000000 0.1034838 1.5789848 1.7823585 2.9816233 1.2284970 1.2961055 0.6203762 0.9423738
0.4006431
Fixed effects: MEASURMENTS ~ as.factor(GROUP)
                       Value Std.Error DF   t-value p-value
(Intercept)         40.30330  3.661662 18  11.00683       0
as.factor(GROUP)DN -12.82013  0.804876 18 -15.92808       0
 Correlation:
                   (Intr)
as.factor(GROUP)DN -0.092

Standardized Within-Group Residuals:
         Min           Q1          Med           Q3          Max
-1.281830598 -0.695807847 -0.006646045  0.768642613  1.619162874

Number of Observations: 29
Number of Groups: 10
```

Weight Gain Week 12

- Separate variance for each cage

```
lme(MEASURMENTS~as.factor(GROUP),na.action=na.exclude,random=~1|CAGE,weights=varIdent(form=~1|
CAGE))

Linear mixed-effects model fit by REML

       AIC      BIC    logLik
  262.3421 279.188 -118.1711

Random effects:
 Formula: ~1 | CAGE
        (Intercept) Residual
StdDev:    19.09569 1.840794

Variance function:
 Structure: Different standard deviations per stratum
 Formula: ~1 | CAGE
 Parameter estimates:
        5         6         7         8         9        10        11        12        13
14
 1.000000  4.844124 11.841168 13.314933 11.952929  7.701824  8.327383  3.637733 15.390009
5.920469
Fixed effects: MEASURMENTS ~ as.factor(GROUP)
                       Value Std.Error DF   t-value p-value
(Intercept)         80.74312  6.849613 18  11.78798       0
as.factor(GROUP)DN -25.93712  2.048303 18 -12.66274       0
 Correlation:
                   (Intr)
as.factor(GROUP)DN -0.129

Standardized Within-Group Residuals:
       Min         Q1        Med         Q3        Max
-1.4987695 -0.7199205  0.1069572  0.6272421  1.6227194

Number of Observations: 29
Number of Groups: 10
```

Adipocyte size

- Separate variance for each cage

```
lme(MEASURMENTS~as.factor(GROUP),na.action=na.exclude,random=~1|CAGE,weights=varIdent(form=~1|
CAGE))

Linear mixed-effects model fit by REML


      AIC      BIC   logLik
  417.096 431.7225 -196.548


Random effects:
 Formula: ~1 | CAGE
        (Intercept) Residual
StdDev:    205.8004 553.1374


Variance function:
 Structure: Different standard deviations per stratum
 Formula: ~1 | CAGE
 Parameter estimates:
         4          5          6          7          8          9         10         11
12
1.00000000 0.07704862 0.33401133 1.61261520 2.78493998 0.61984231 1.71281120 0.27927668
2.60268312
Fixed effects: MEASURMENTS ~ as.factor(GROUP)
                      Value Std.Error DF   t-value p-value
(Intercept)        4526.546 109.96148 17  41.16483       0
as.factor(GROUP)DN -508.252  48.61002 17 -10.45571       0
 Correlation:
                   (Intr)
as.factor(GROUP)DN -0.218


Standardized Within-Group Residuals:
        Min          Q1         Med          Q3         Max
-1.96562200 -0.58563257  0.07957057  0.64274551  1.64166232


Number of Observations: 27
Number of Groups: 9
```

Liver Steatosis

```
lme(MEASURMENTS~as.factor(GROUP),na.action=na.exclude,random=~1|CAGE)
Linear mixed-effects model fit by REML

       AIC      BIC    logLik
  170.5876 175.1296 -81.29379

Random effects:
 Formula: ~1 | CAGE
        (Intercept) Residual
StdDev:    3.769916 6.706968

Fixed effects: MEASURMENTS ~ as.factor(GROUP)
                       Value Std.Error DF  t-value p-value
(Intercept)        27.225011  2.444700 16 11.13634  0.0000
as.factor(GROUP)DN -4.896344  2.738187 16 -1.78817  0.0927
 Correlation:
                   (Intr)
as.factor(GROUP)DN -0.629

Standardized Within-Group Residuals:
       Min         Q1        Med         Q3        Max
-2.5428596 -0.3756805  0.2148794  0.5911253  1.2486900

Number of Observations: 25
Number of Groups: 8
```

Liver Srebp1c mRNA

- Separate variance for each cage

```
lme(MEASURMENTS~as.factor(GROUP),na.action=na.exclude,random=~1|CAGE,weights=varIdent(form=~1|
CAGE))

Linear mixed-effects model fit by REML


       AIC      BIC    logLik
  128.8651 140.8665 -53.43253


Random effects:
 Formula: ~1 | CAGE
        (Intercept) Residual
StdDev:   0.5253005 3.495393


Variance function:
 Structure: Different standard deviations per stratum
 Formula: ~1 | CAGE
 Parameter estimates:
        4         5         6         7         8        10        11        12
1.0000000 0.2624571 0.5421273 0.1554060 1.1049000 0.4750143 2.5899755 1.1895210
Fixed effects: MEASURMENTS ~ as.factor(GROUP)
                       Value Std.Error DF   t-value p-value
(Intercept)         9.298797 0.4697197 15 19.796480   0.000
as.factor(GROUP)DN -1.874583 0.4590505 15 -4.083609   0.001
 Correlation:
                   (Intr)
as.factor(GROUP)DN -0.554


Standardized Within-Group Residuals:
       Min         Q1        Med         Q3        Max
-1.3887363 -0.4700683  0.2445098  0.8079815  1.8443333


Number of Observations: 24
Number of Groups: 8
```

Liver Acaca mRNA

```
lme(MEASURMENTS~as.factor(GROUP),na.action=na.exclude,random=~1|CAGE)
Linear mixed-effects model fit by REML

       AIC      BIC    logLik
  136.9992 141.8747 -64.49961

Random effects:
 Formula: ~1 | CAGE
        (Intercept) Residual
StdDev:    2.417757 2.254051

Fixed effects: MEASURMENTS ~ as.factor(GROUP)
                       Value Std.Error DF   t-value p-value
(Intercept)         7.195587 0.9827833 16  7.321641  0.0000
as.factor(GROUP)DN -1.988653 0.9206586 16 -2.160033  0.0463
 Correlation:
                   (Intr)
as.factor(GROUP)DN -0.423

Standardized Within-Group Residuals:
       Min         Q1        Med         Q3        Max
-1.5057243 -0.6889085  0.1725828  0.4741543  1.8815818

Number of Observations: 27
Number of Groups: 10
```

Liver Fasn mRNA

```
lme(MEASURMENTS~as.factor(GROUP),na.action=na.exclude,random=~1|CAGE)
Linear mixed-effects model fit by REML

       AIC      BIC    logLik
  79.85878 84.73428 -35.92939

Random effects:
 Formula: ~1 | CAGE
        (Intercept) Residual
StdDev:   0.3574923 0.858634

Fixed effects: MEASURMENTS ~ as.factor(GROUP)
                        Value Std.Error DF   t-value p-value
(Intercept)         2.0619022 0.2579059 16  7.994786  0.0000
as.factor(GROUP)DN -0.6705682 0.3379362 16 -1.984304  0.0646
 Correlation:
                   (Intr)
as.factor(GROUP)DN -0.618

Standardized Within-Group Residuals:
       Min         Q1        Med         Q3        Max
-1.4592421 -0.7099803 -0.2408227  0.3804016  2.2347082

Number of Observations: 27
Number of Groups: 10
```

Liver Scd1 mRNA

```
lme(MEASURMENTS~as.factor(GROUP),na.action=na.exclude,random=~1|CAGE)
Linear mixed-effects model fit by REML

       AIC      BIC    logLik
  117.8604 122.7359 -54.93018

Random effects:
 Formula: ~1 | CAGE
        (Intercept) Residual
StdDev:    0.688461 1.857884

Fixed effects: MEASURMENTS ~ as.factor(GROUP)
                       Value Std.Error DF   t-value p-value
(Intercept)         3.924979 0.5460840 16  7.187501  0.0000
as.factor(GROUP)DN -1.067380 0.7286659 16 -1.464841  0.1623
 Correlation:
                   (Intr)
as.factor(GROUP)DN -0.631

Standardized Within-Group Residuals:
       Min         Q1        Med         Q3        Max
-1.4126724 -0.4593962 -0.1350479  0.4058632  2.3005514

Number of Observations: 27
Number of Groups: 10
```

CHOLESTEROL

```
lme(MEASURMENTS~as.factor(GROUP),na.action=na.exclude,random=~1|CAGE)
Linear mixed-effects model fit by REML

       AIC     BIC    logLik
  123.4102 127.188 -57.70511

Random effects:
 Formula: ~1 | CAGE
        (Intercept) Residual
StdDev:   0.6351555 4.421418

Fixed effects: MEASURMENTS ~ as.factor(GROUP)
                       Value Std.Error DF   t-value p-value
(Intercept)        22.517433  1.583070 12 14.223904   0.000
as.factor(GROUP)DN -1.466909  1.989032 12 -0.737499   0.475
 Correlation:
                   (Intr)
as.factor(GROUP)DN -0.779

Standardized Within-Group Residuals:
        Min          Q1         Med          Q3         Max
-2.86810542 -0.50231786  0.08201051  0.46205784  2.25844724

Number of Observations: 21
Number of Groups: 8
```

HDL

```
lme(MEASURMENTS~as.factor(GROUP),na.action=na.exclude,random=~1|CAGE)
Linear mixed-effects model fit by REML

        AIC       BIC   logLik
  -28.40044 -24.83895 18.20022

Random effects:
 Formula: ~1 | CAGE
        (Intercept)   Residual
StdDev:  0.03013001 0.07249113

Fixed effects: MEASURMENTS ~ as.factor(GROUP)
                        Value  Std.Error DF   t-value p-value
(Intercept)         0.3535842 0.02813218 11 12.568674  0.0000
as.factor(GROUP)DN -0.0010577 0.03332860 11 -0.031736  0.9753
 Correlation:
                   (Intr)
as.factor(GROUP)DN -0.716

Standardized Within-Group Residuals:
       Min         Q1        Med         Q3        Max
-0.8908851 -0.5403010 -0.4401045  0.5663041  1.9694419

Number of Observations: 20
Number of Groups: 8
```

LDL

```
lme(MEASURMENTS~as.factor(GROUP),na.action=na.exclude,random=~1|CAGE)
Linear mixed-effects model fit by REML

       AIC      BIC    logLik
  104.6889 108.2503 -48.34443

Random effects:
 Formula: ~1 | CAGE
        (Intercept) Residual
StdDev:    1.638636 2.781935

Fixed effects: MEASURMENTS ~ as.factor(GROUP)
                       Value Std.Error DF   t-value p-value
(Intercept)        21.291422  1.163471 11 18.299918  0.0000
as.factor(GROUP)DN -0.416924  1.284565 11 -0.324564  0.7516
 Correlation:
                   (Intr)
as.factor(GROUP)DN -0.67

Standardized Within-Group Residuals:
       Min         Q1        Med         Q3        Max
-1.4513596 -0.5360393  0.1487976  0.4505872  2.5088054

Number of Observations: 20
Number of Groups: 8
```

TRIGLYCERIDES

- No random intercept

```
lm(MEASURMENTS~as.factor(GROUP),na.action=na.exclude)
Call:
lm(formula = MEASURMENTS ~ as.factor(GROUP),
    na.action = na.exclude)

Residuals:
    Min      1Q  Median      3Q     Max
-1.2286 -0.3000  0.0000  0.2714  1.3714

Coefficients:
                   Estimate Std. Error t value Pr(>|t|)
(Intercept)          2.2000     0.1681  13.088 3.84e-12 ***
as.factor(GROUP)DN  -0.3714     0.2246  -1.654    0.112
---
Signif. codes:  0 '***' 0.001 '**' 0.01 '*' 0.05 '.' 0.1 ' ' 1

Residual standard error: 0.5575 on 23 degrees of freedom
  (10 observations deleted due to missingness)
Multiple R-squared:  0.1062,    Adjusted R-squared:  0.06739
F-statistic: 2.734 on 1 and 23 DF,  p-value: 0.1118
```
